# Supplementary material for: Lasianosides A–E: New Iridoid Glucosides from the Leaves of Lasianthus verticillatus (Lour.) Merr. and Their Antioxidant Activity
Source: Molecules. 2019 Nov 5;24(21):3995. doi: 10.3390/molecules24213995 (PMC6864479; doi:10.3390/molecules24213995)
Supplement: Supplementary file 1 [file molecules-24-03995-s001.pdf]

Spectral Data

Lasianosides A-E: iridoid glucosides from the aerial parts of *Lasianthus verticillatus* (Lour.) Merr.

Gadah Al-Hamoud[1,2], Raha Saud Orfali[2], Shagufta Perveen, Kenta Mizuno[3], Yoshio Takeda[3], Tatsuo Nehira[4], Kazuma Masuda[4], Sachiko Sugimoto[1], Yoshi Yamano[1], Hideaki Otsuka[1,5], Katsuyoshi Matsunami[1,*]

1. Graduate School of Biomedical and Health Sciences, Hiroshima University, 1-2-3 Kasumi, Minami-ku, Hiroshima 734-8553

2 Department of Pharmacognosy, College of Pharmacy, King Saud University, Riyadh 11495, Kingdom of Saudi Arabia

3 Faculty of Integrated Arts and Sciences, The University of Tokushima, 1-1 Minamijosanjima-Cho, Tokushima 770-8502, Japan

4 Graduate School of Integrated Arts and Sciences, Hiroshima University, 1-7-1 Kagamiyama, Higashi-Hiroshima 739-8521, Japan

5 Faculty of Pharmacy, Yasuda Women's University, 6-13-1 Yasuhigashi, Asaminami-ku, Hiroshima 731-0153, Japan

Contents:

Figure 1. The HRESIMS spectrum of **1**.
Figure 2. The ^{1}H NMR spectrum of **1** in CD_3OD, 500 MHz.
Figure 3. The ^{13}C NMR spectrum of **1** in CD_3OD, 175 MHz.
Figure 4. The DEPT-135 NMR spectrum of **1** in CD_3OD, 175 MHz.
Figure 5. The COSY spectrum of **1** in CD_3OD, 700 MHz.
Figure 6. The HSQC spectrum of **1** in CD_3OD, 700 MHz.
Figure 7. The HMBC spectrum of **1** in CD_3OD, 700 MHz.
Figure 8. The NOESY spectrum of **1** in CD_3OD, 500 MHz.
Figure 9. The IR spectrum of **1**.
Figure 10. The HRESIMS spectrum of **2**.
Figure 11. The ^{1}H NMR spectrum of **2** in CD_3OD, 500 MHz.
Figure 12. The ^{13}C NMR spectrum of **2** in CD_3OD, 175 MHz.
Figure 13. The DEPT-135 NMR spectrum of **2** in CD_3OD, 175 MHz.
Figure 14. The COSY spectrum of **2** in CD_3OD, 700 MHz.
Figure 15. The HSQC spectrum of **2** in CD_3OD, 700 MHz.
Figure 16. The HMBC spectrum of **2** in CD_3OD, 700 MHz.
Figure 17. The NOESY spectrum of **2** in CD_3OD, 500 MHz.
Figure 18. The IR spectrum of **2**.
Figure 19. The HRESIMS spectrum of **3**.
Figure 20. The ^{1}H NMR spectrum of **3** in CD_3OD, 500 MHz.
Figure 21. The ^{13}C NMR spectrum of **3** in CD_3OD, 175 MHz.
Figure 22. The DEPT-135 NMR spectrum of **3** in CD_3OD, 175 MHz
Figure 23. The COSY spectrum of **3** in CD_3OD, 700 MHz
Figure 24. The HSQC spectrum of **3** in CD_3OD, 700 MHz.
Figure 25. The HMBC spectrum of **3** in CD_3OD, 700 MHz
Figure 26. The NOESY spectrum of **3** in CD_3OD, 500 MHz
Figure 27. The IR spectrum of **3**.
Figure 28. The UV spectrum of **3**.
Figure 29. The HRESIMS spectrum of **4**.
Figure 30. The ^{1}H NMR spectrum of **4** in CD_3OD, 500 MHz.
Figure 31. The ^{13}C NMR spectrum of **4** in CD_3OD, 175 MHz.
Figure 32. The DEPT-135 NMR spectrum of **4** in CD_3OD, 175 MHz
Figure 33. The COSY spectrum of **4** in CD_3OD, 500 MHz
Figure 34. The HSQC spectrum of **4** in CD_3OD, 700 MHz.
Figure 35. The HMBC spectrum of **4** in CD_3OD, 700 MHz
Figure 36. The NOESY spectrum of **4** in CD_3OD, 500 MHz
Figure 37. The IR spectrum of **4**.
Figure 38. The UV spectrum of **4**.
Figure 39. The HRESIMS spectrum of **5**.
Figure 40. The ^{1}H NMR spectrum of **5** in CD_3OD, 500 MHz.

Figure 41. The ^{13}C NMR spectrum of **5** in CD_3OD, 175 MHz.
Figure 42. The DEPT-135 NMR spectrum of **5** in CD_3OD, 175 MHz
Figure 43. The COSY spectrum of **5** in CD_3OD, 500 MHz
Figure 44. The HSQC spectrum of **5** in CD_3OD, 700 MHz.
Figure 45. The HMBC spectrum of **5** in CD_3OD, 700 MHz
Figure 46. The NOESY spectrum of **5** in CD_3OD, 500 MHz
Figure 47. The IR spectrum of **5**.
Figure 48. The UV spectrum of **5**.

Figure 1. The HRESIMS spectrum of **1**.

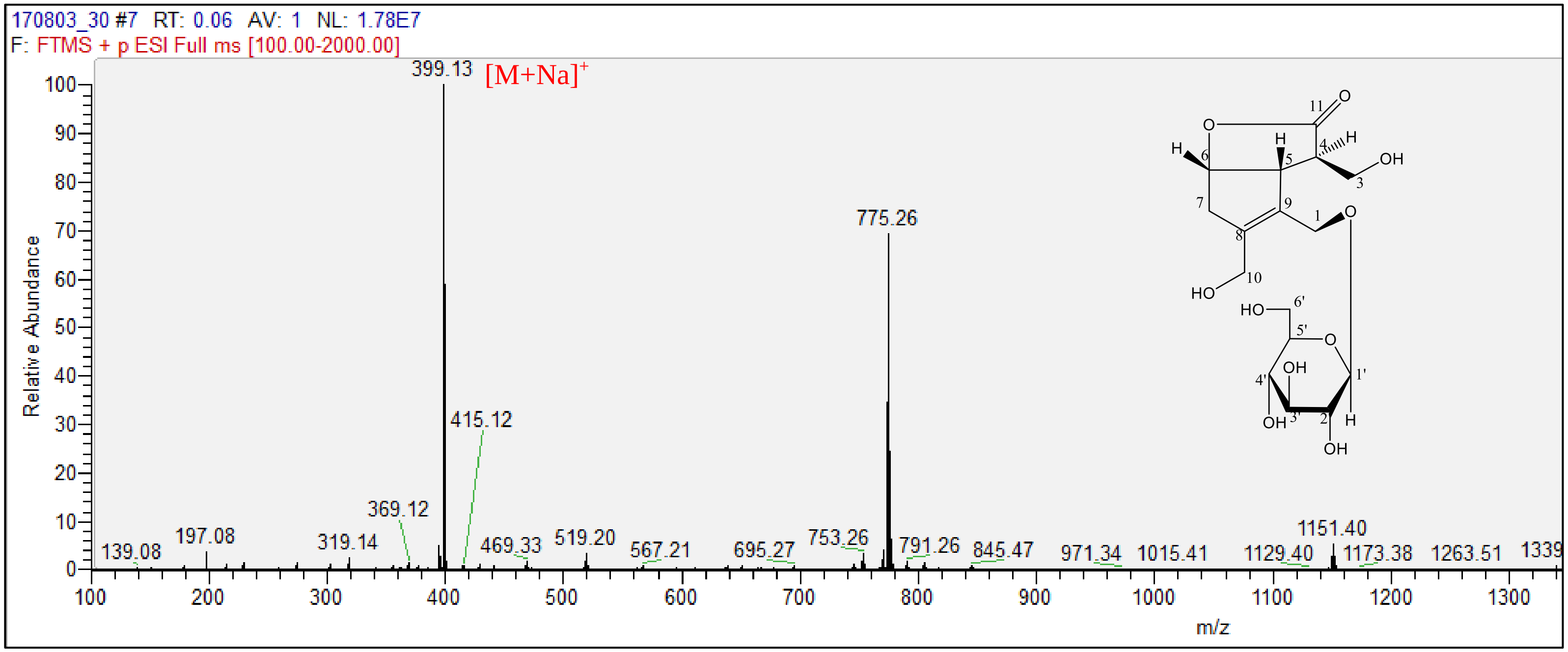

Elemental composition search on mass 399.13

m/z= 394.13-404.13

m/z	Theo. Mass	Delta (mmu)	RDB equiv.	Composition
399.1263	399.1262	0.17	4.5	$C_{16}H_{24}O_{10}Na$

Figure 2. The ^{1}H NMR spectrum of **1** in CD_3OD, 500 MHz.

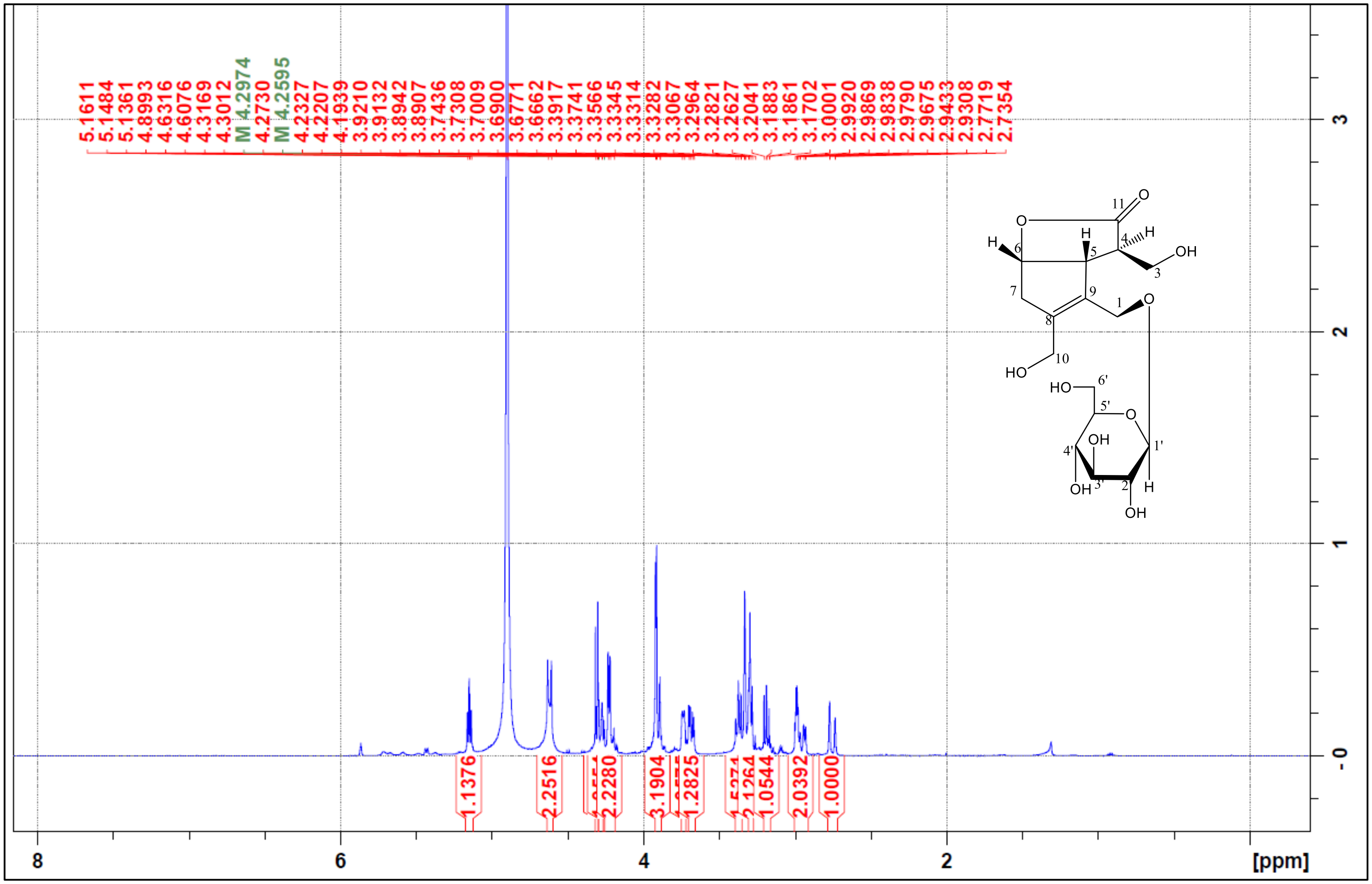

Figure 3. The ^{13}C NMR spectrum of **1** in CD_3OD, 175 MHz.

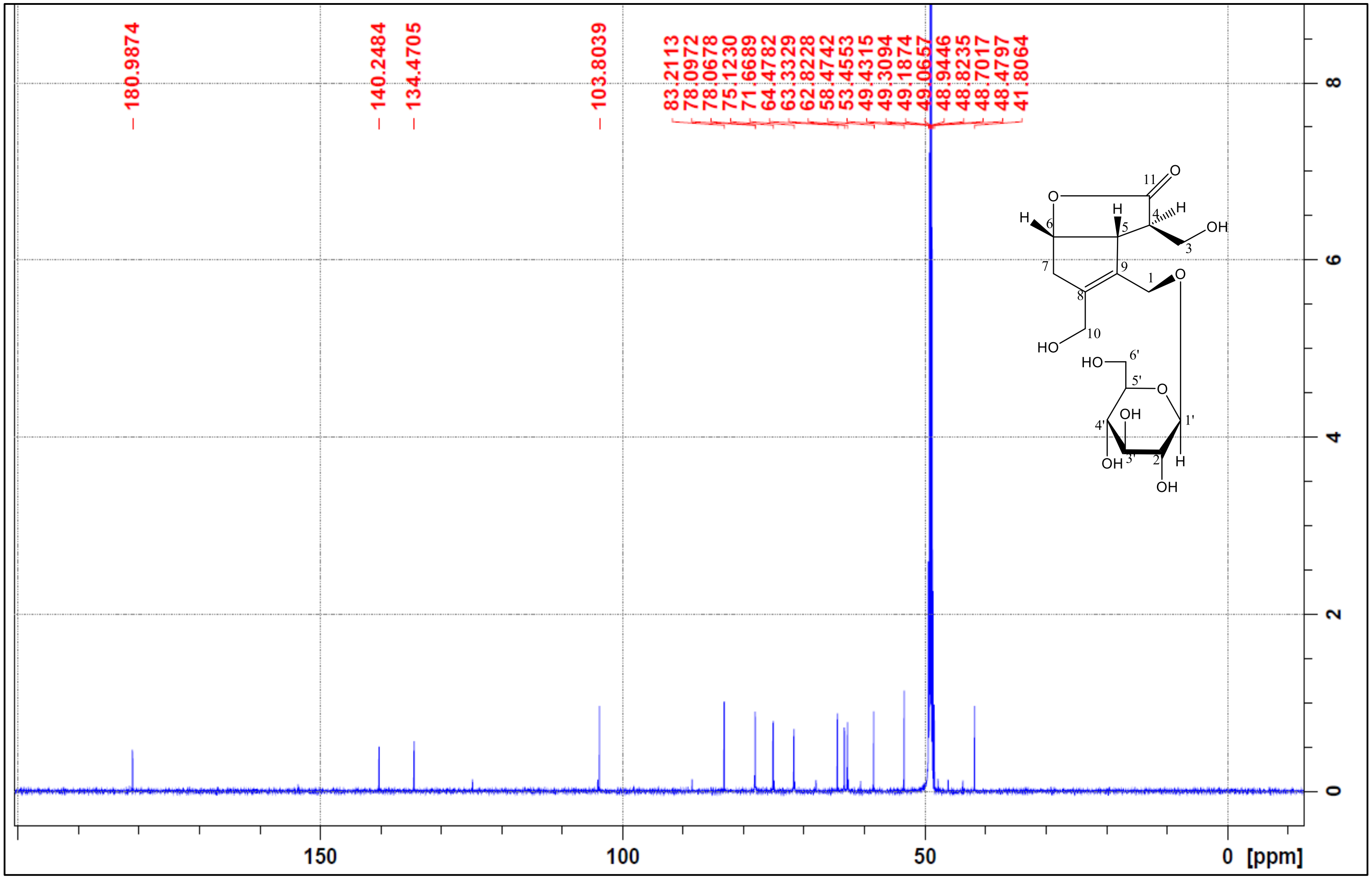

Figure 4. The DEPT-135 NMR spectrum of **1** in CD_3OD, 175 MHz.

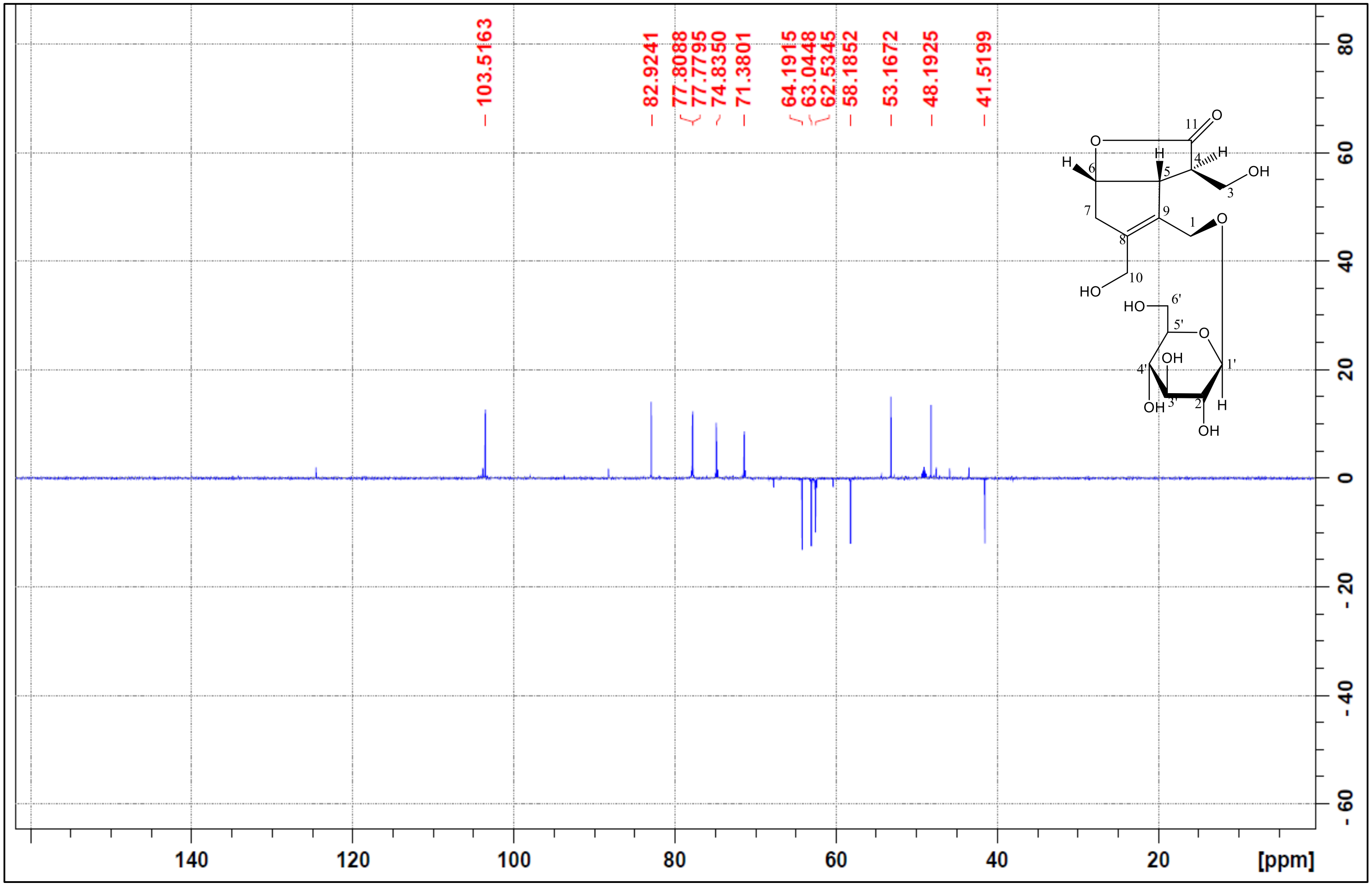

Figure 5. The COSY spectrum of **1** in CD_3OD, 700 MHz.

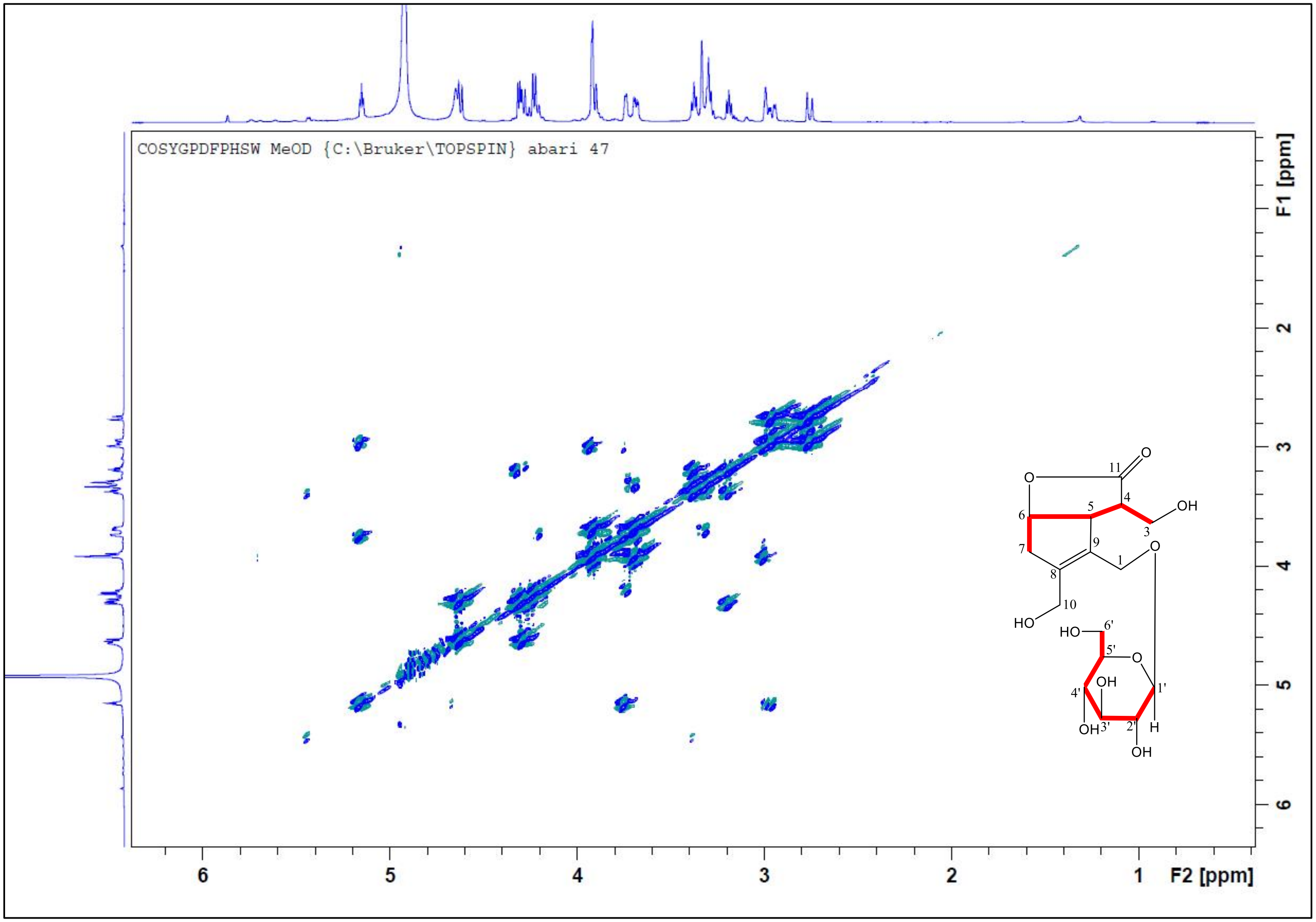

Figure 6. The HSQC spectrum of **1** in CD_3OD, 700 MHz.

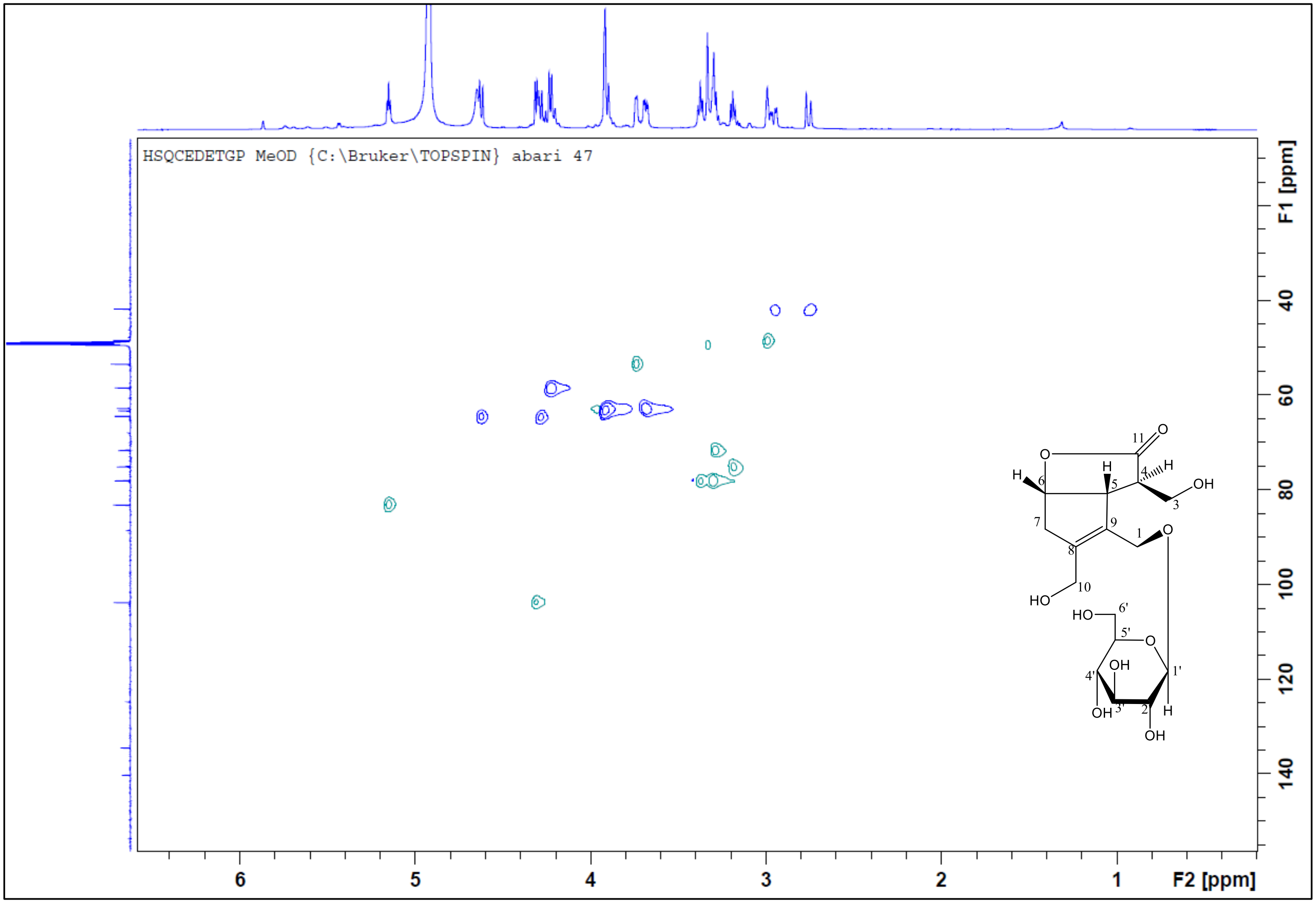

Figure 7. The HMBC spectrum of **1** in CD_3OD, 700 MHz.

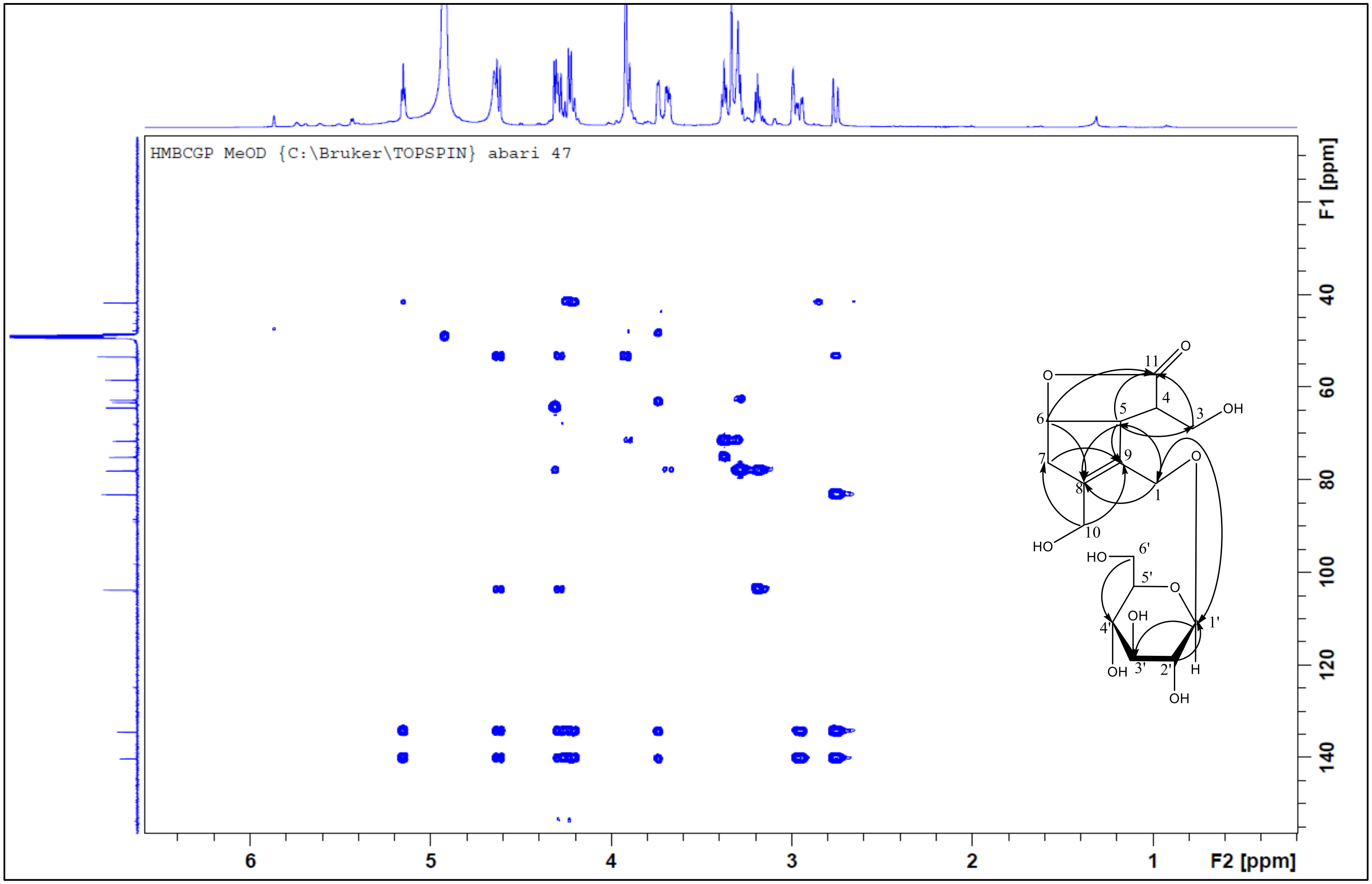

Figure 8. The NOESY spectrum of **1** in CD_3OD, 500 MHz.

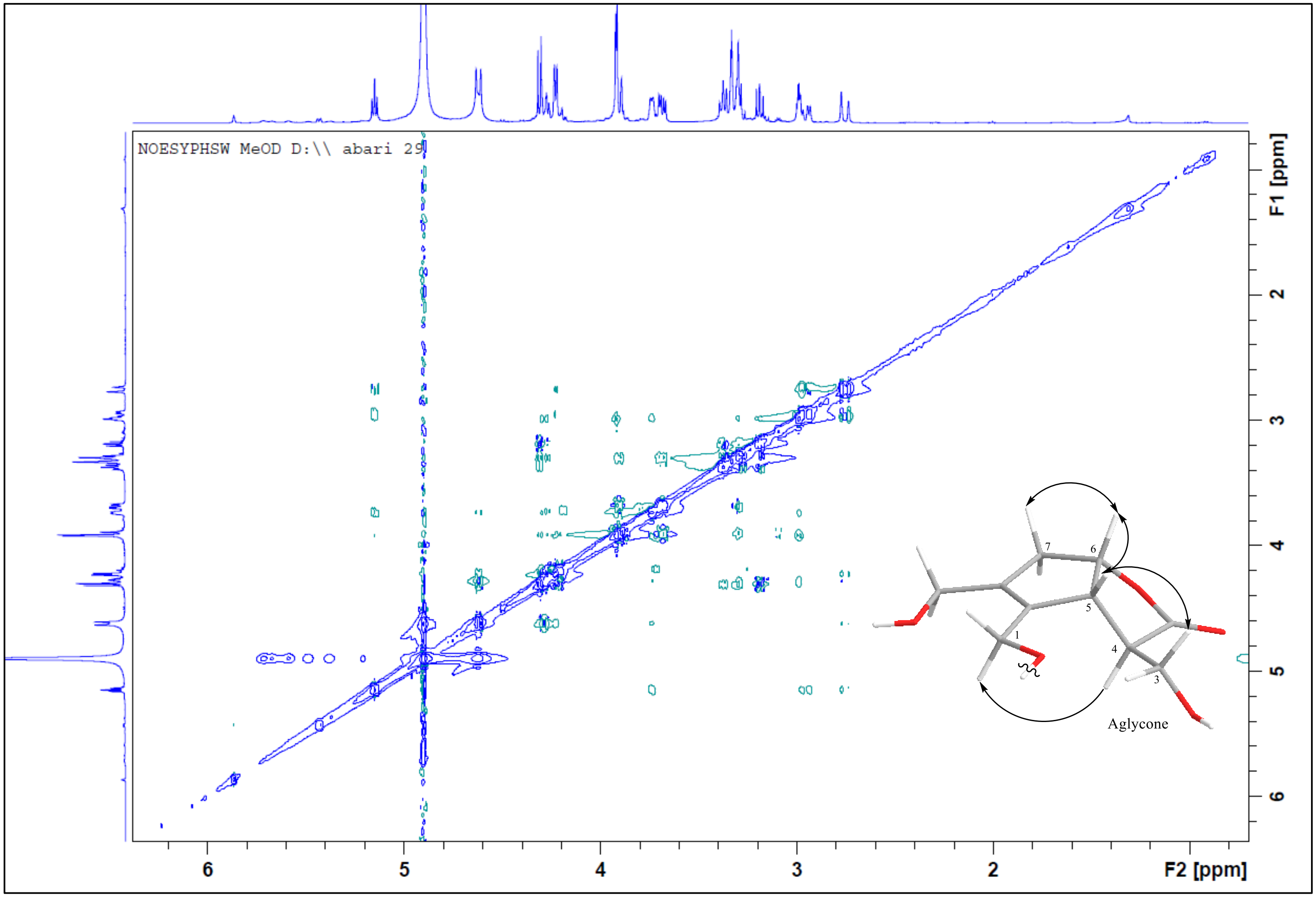

Figure 9. The IR spectrum of **1**.

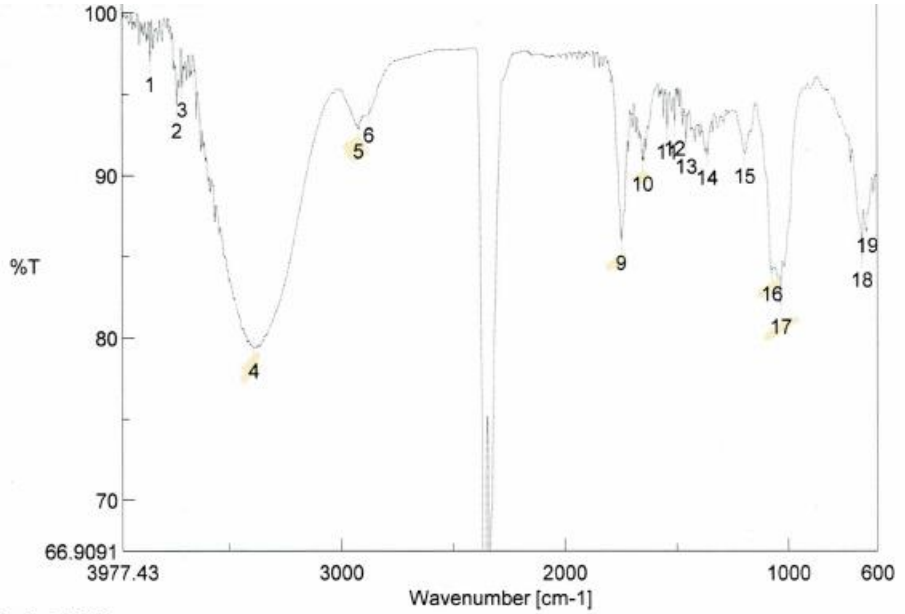

[コメント情報]
試料名
コメント
測定者
所属
会社 HU

[データ情報]		[測定情報]	
作成日時	2018/06/28 17:32	機種名	FT/IR-4600typeA
		シリアル番号	D015461786
データタイプ	等間隔データ		
横軸	Wavenumber [cm-1]	測定日時	2018/06/28 17:32
縦軸	%T		
スタート	599.753 cm-1	光源	標準光源
エンド	7800.65 cm-1	検出器	TGS
データ間隔	0.964233 cm-1	積算回数	10
データ数	7469	分解	4 cm-1
		ゼロフィリング	On
		アポダイゼーション	Cosine
		ゲイン	Auto (2)
		アパーチャー	Auto (7.1 mm)
		スキャンスピード	Auto (2 mm/sec)
		フィルタ	Auto (30000 Hz)

[ピーク検出結果]

No.	位置	強度	No.	位置	強度
1	3852.11	97.0169	2	3733.51	94.1194
3	3709.41	95.432	4	3390.24	79.3491
5	2921.63	92.8682	6	2874.38	93.86
7	2362.37	55.5519	8	2340.19	66.0959
9	1748.16	86.0003	10	1651.73	90.8962
11	1540.85	92.8568	12	1507.1	93.0612
13	1455.99	91.9631	14	1362.46	91.2923

[ピーク検出結果]

No.	位置	強度	No.	位置	強度
15	1194.69	91.3814	16	1072.23	84.0904
17	1032.69	82.0846	18	669.178	84.9505
19	646.036	87.0834			

Figure 10. The HRESIMS spectrum of **2**.

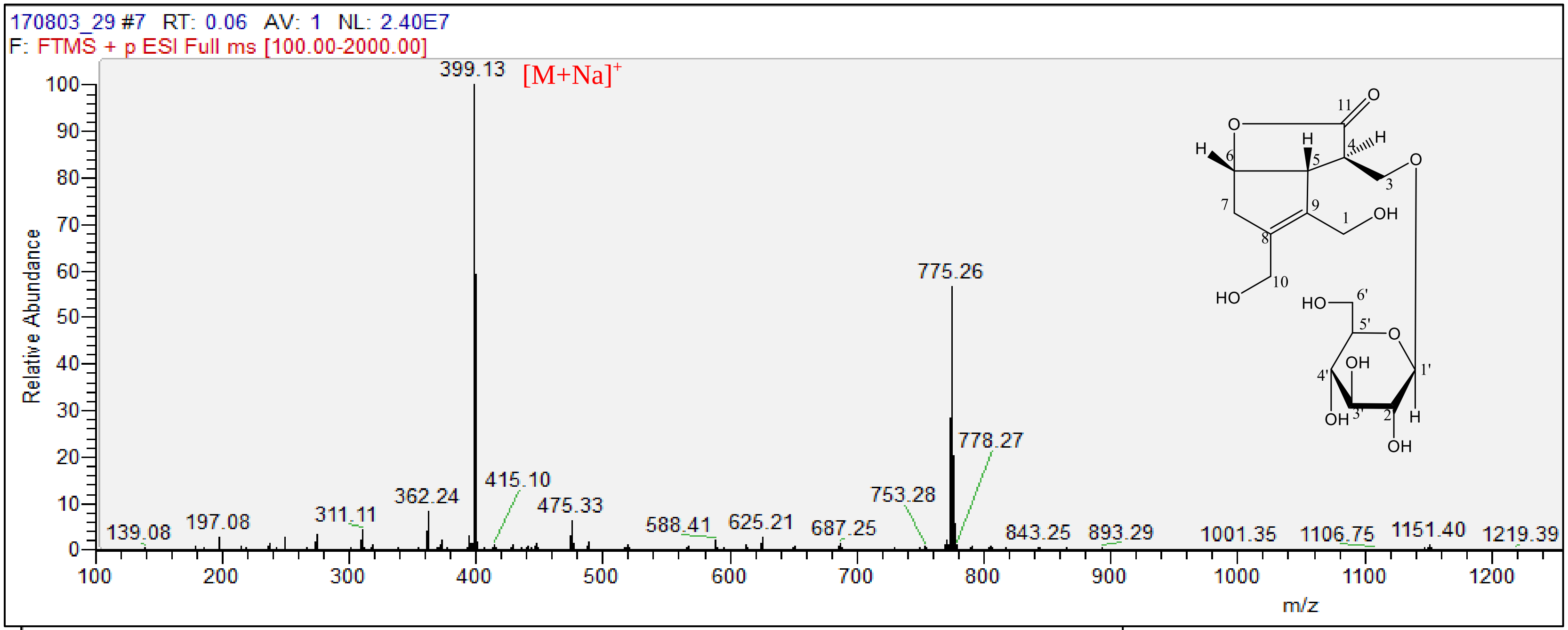

Elemental composition search on mass 399.13

m/z= 394.13-404.13

m/z	Theo. Mass	Delta (mmu)	RDB equiv.	Composition
399.1265	399.1262	0.29	4.5	$C_{16}H_{24}O_{10}Na$

Figure 11. The ^{1}H NMR spectrum of **2** in CD_3OD, 500 MHz.

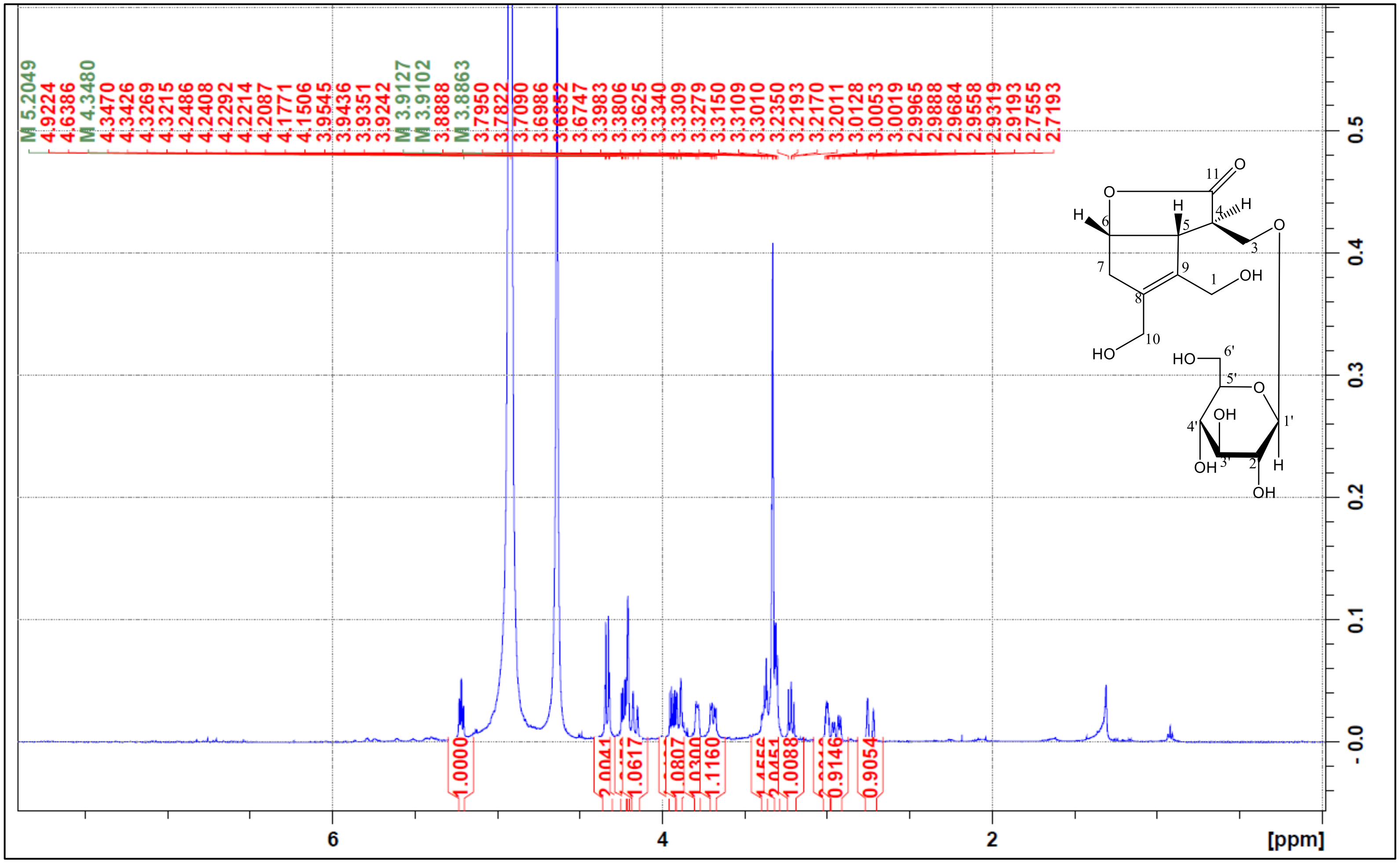

Figure 12. The ^{13}C NMR spectrum of **2** in CD_3OD, 175 MHz.

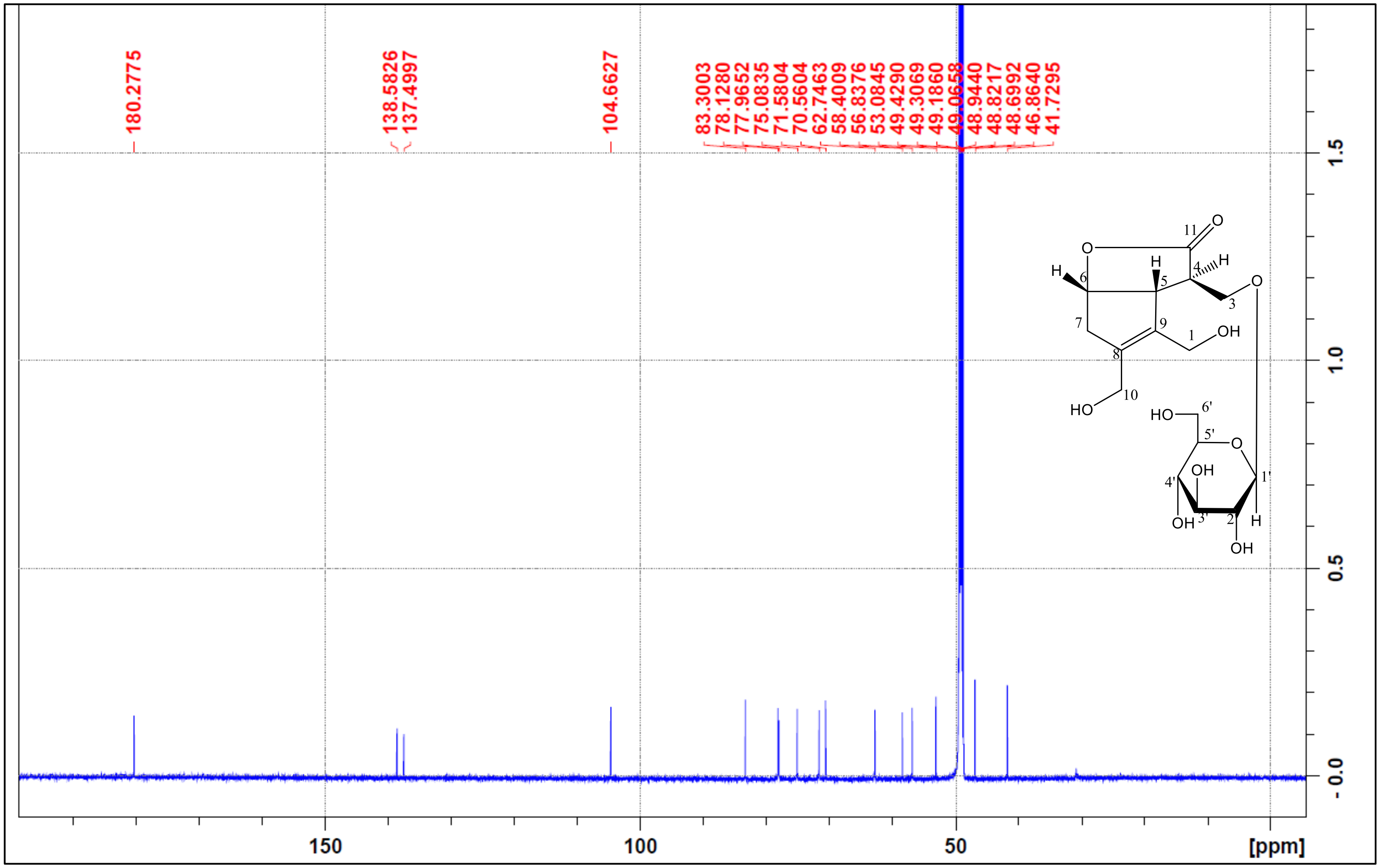

Figure 13. The DEPT-135 NMR spectrum of **2** in CD_3OD, 175 MHz.

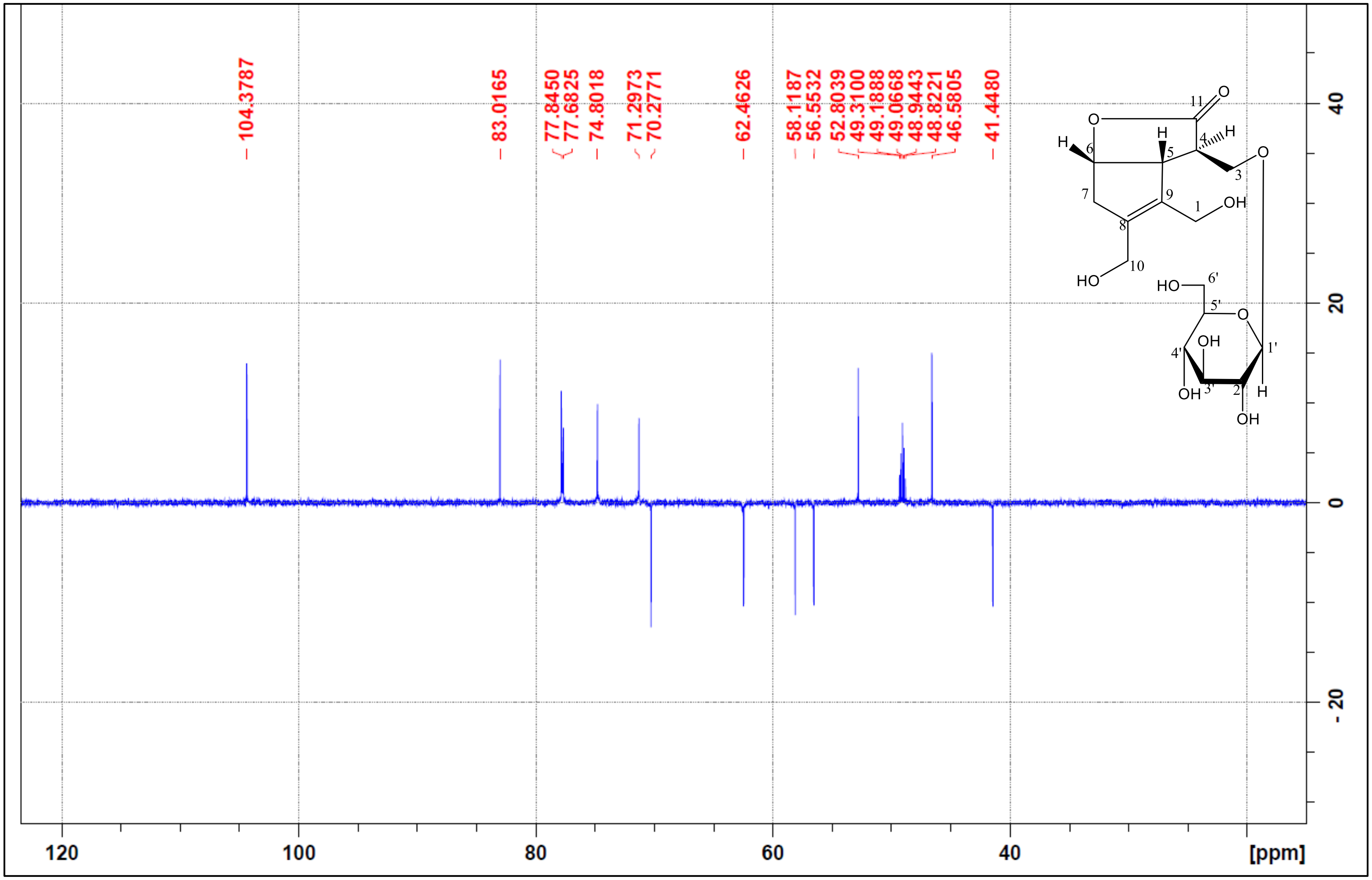

Figure 14. The COSY spectrum of **2** in CD_3OD, 700 MHz.

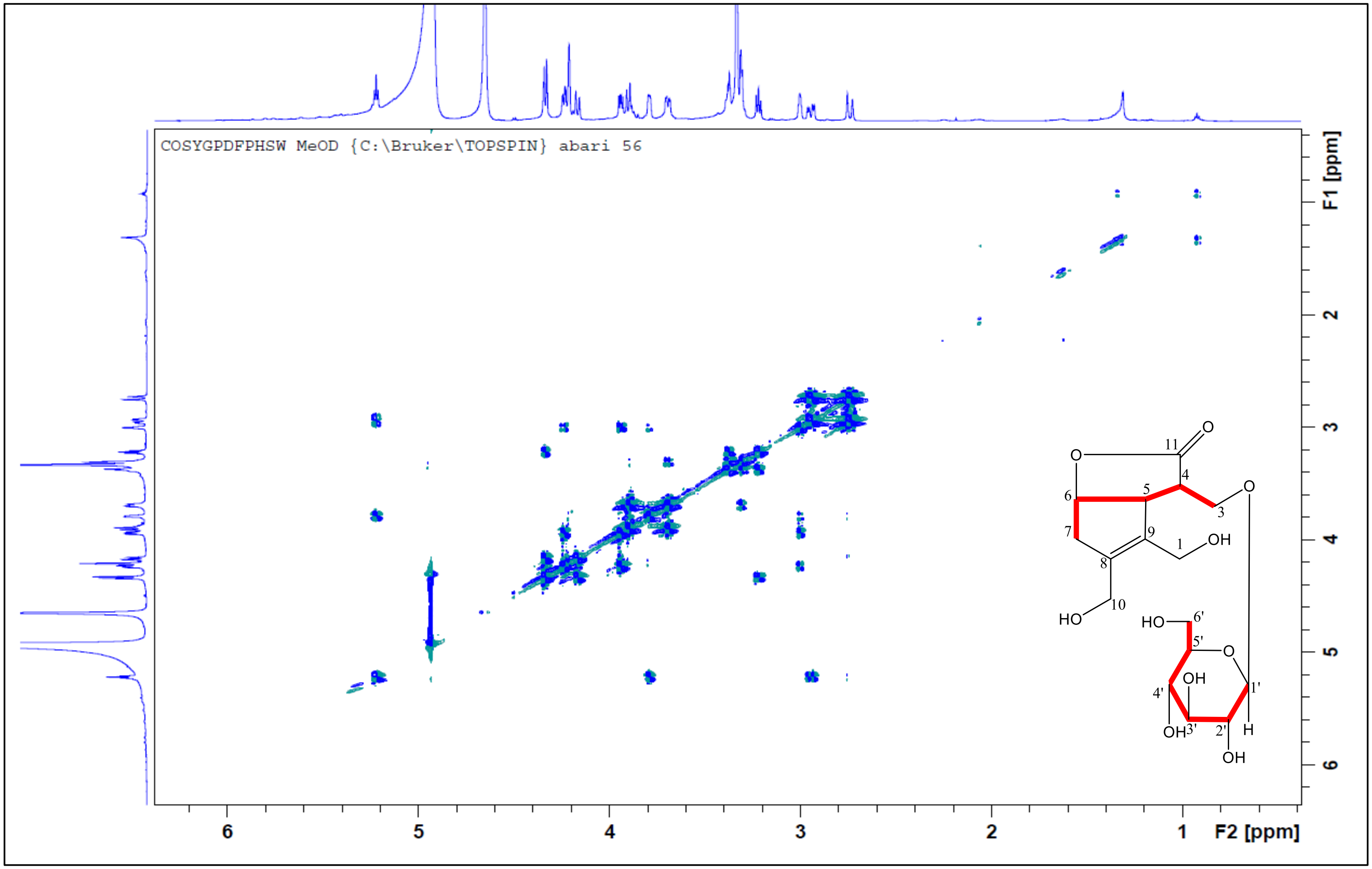

Figure 15. The HSQC spectrum of **2** in CD_3OD, 700 MHz.

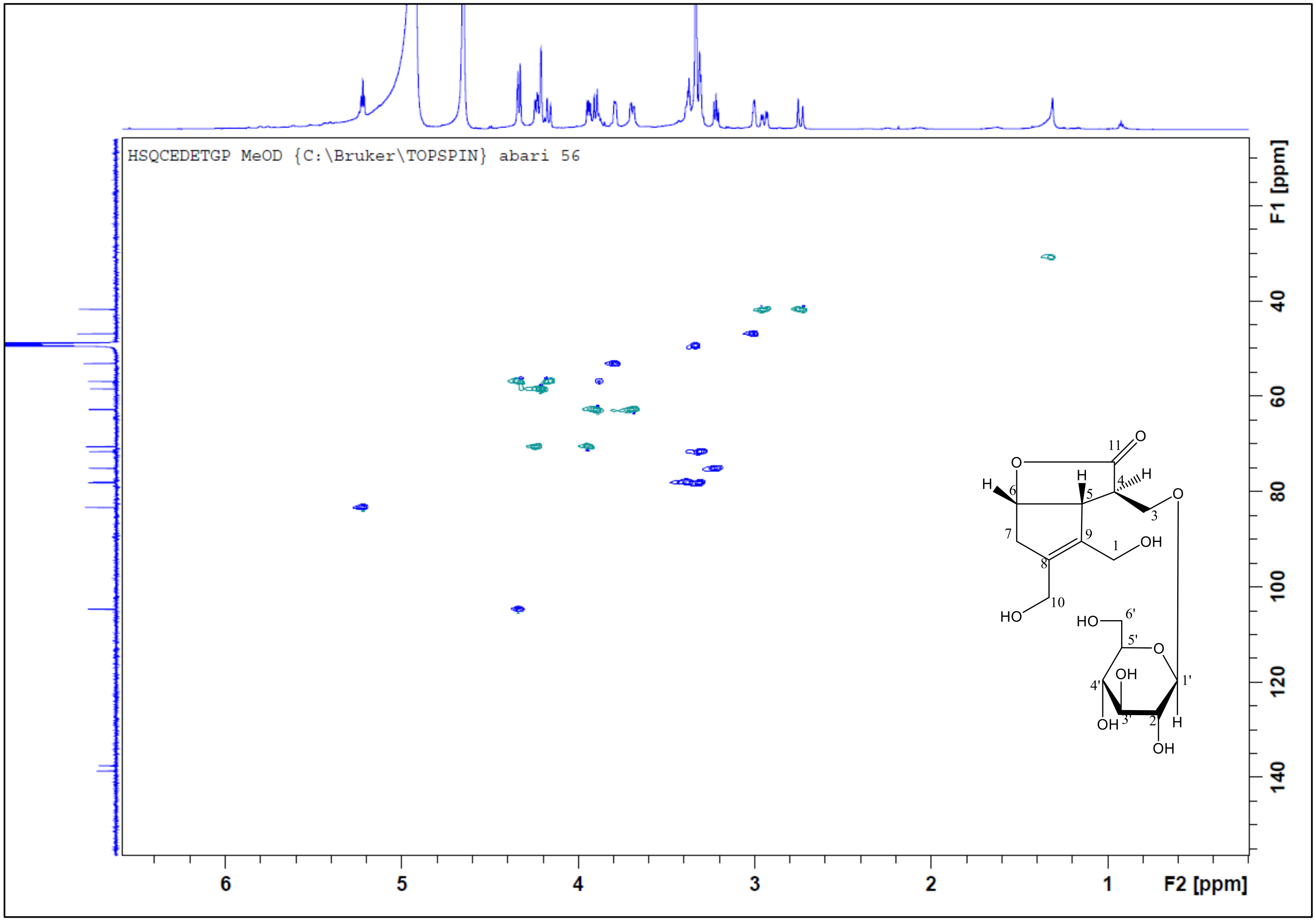

Figure 16. The HMBC spectrum of **2** in CD_3OD, 700 MHz.

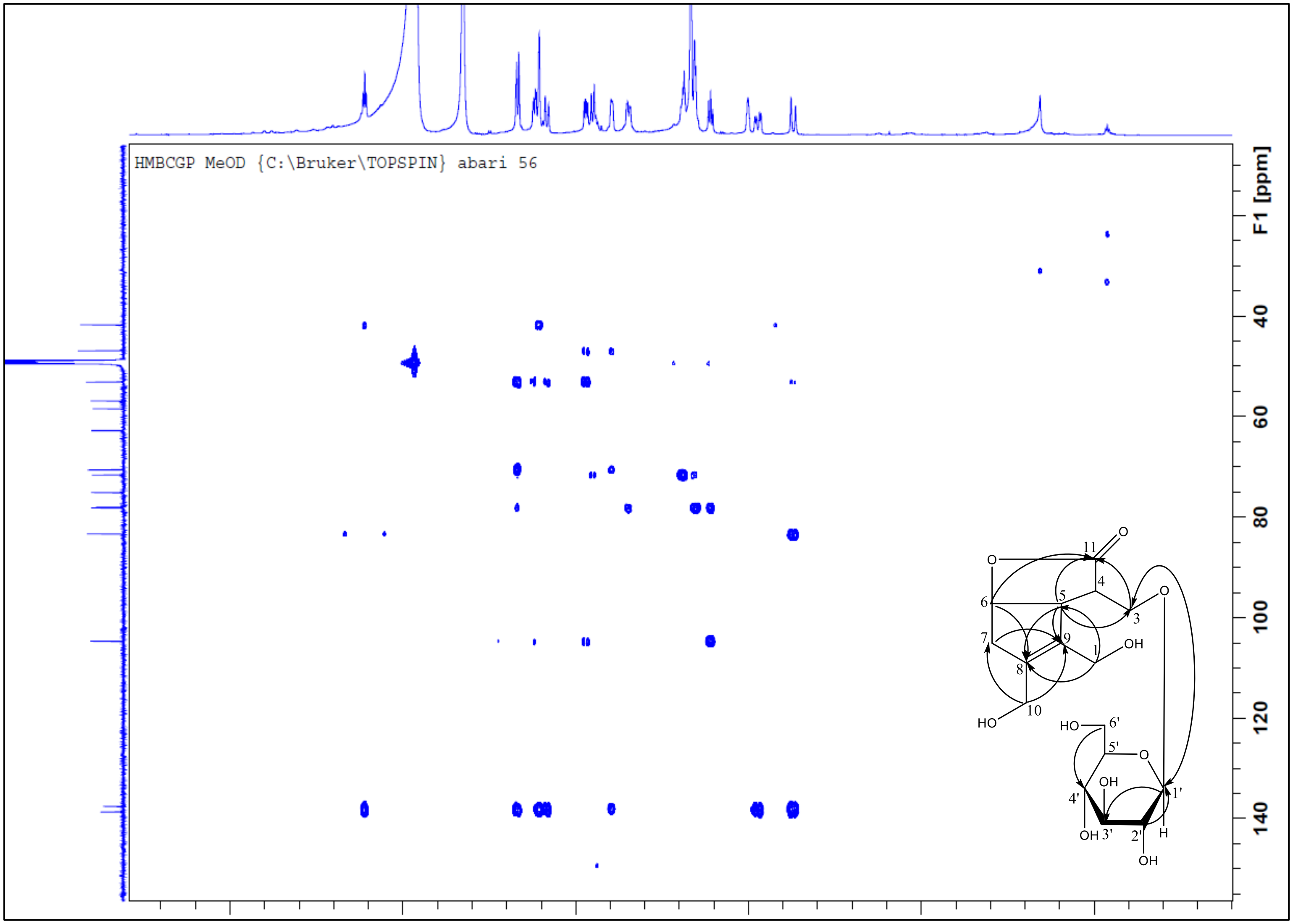

Figure 17. The NOESY spectrum of **2** in CD_3OD, 500 MHz.

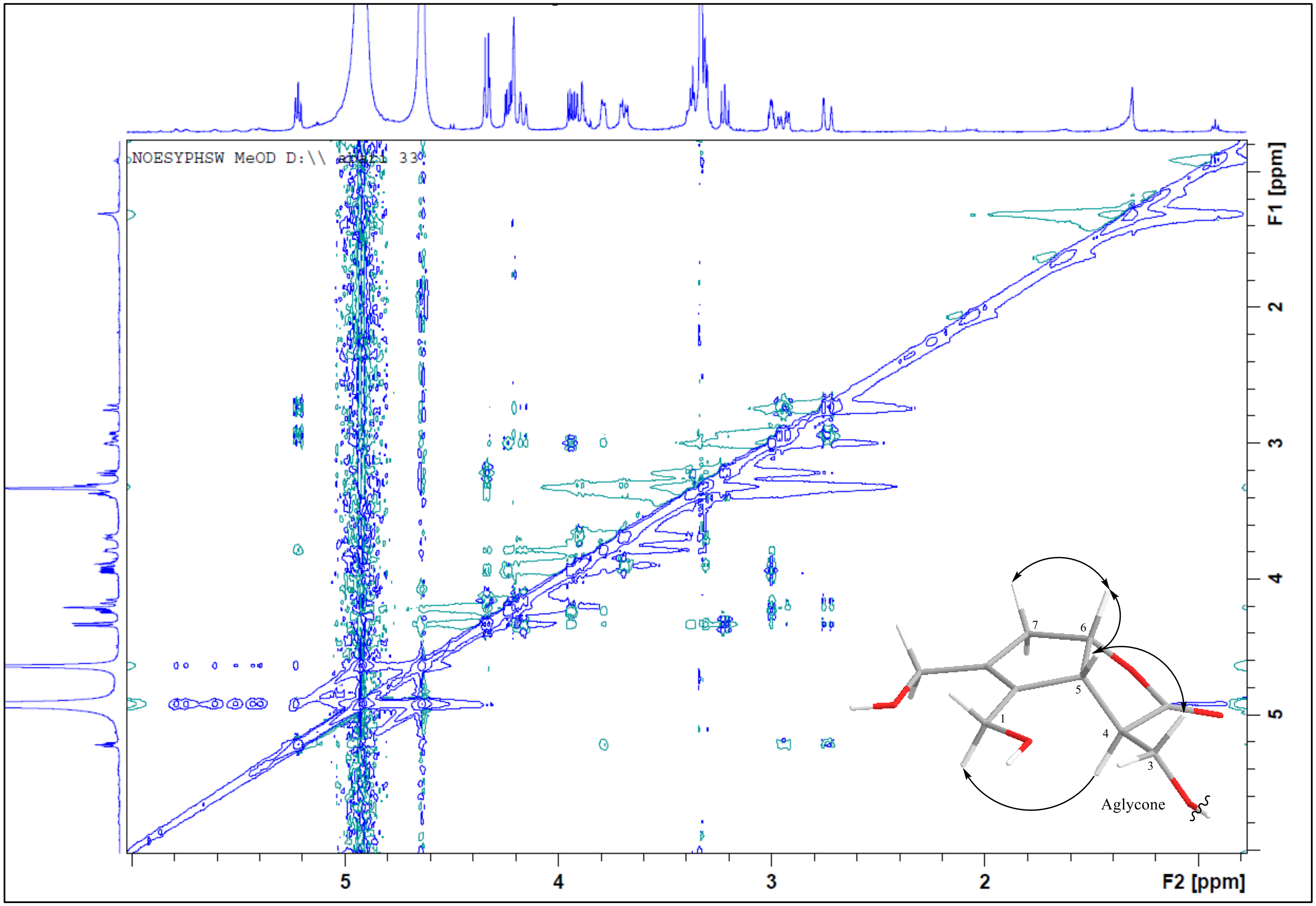

Figure 18. The IR spectrum of **2**.

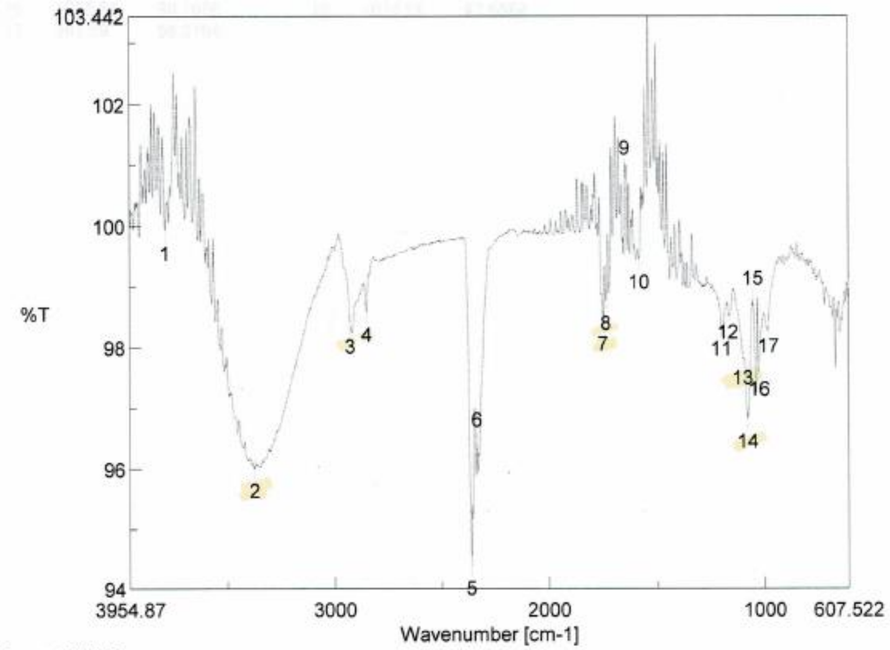

[コメント情報]

試料名	
コメント	
測定者	
所属	
会社	HU

[データ情報]		[測定情報]	
作成日時	2018/07/04 16:22	機種名	FT/IR-4600typeA
		シリアル番号	D015461786
データタイプ	等間隔データ		
横軸	Wavenumber [cm-1]	測定日時	2018/07/04 16:22
縦軸	%T		
スタート	599.753 cm-1	光源	標準光源
エンド	7800.65 cm-1	検出器	TGS
データ間隔	0.964233 cm-1	積算回数	10
データ数	7469	分解	4 cm-1
		ゼロフィリング	On
		アポダイゼーション	Cosine
		ゲイン	Auto (2)
		アパーチャー	Auto (7.1 mm)
		スキャンスピード	Auto (2 mm/sec)
		フィルタ	Auto (30000 Hz)

[ピーク検出結果]

No.	位置	強度	No.	位置	強度
1	3790.4	99.915	2	3372.89	95.9975
3	2927.41	98.3722	4	2850.27	98.5695
5	2363.34	94.3719	6	2337.3	96.4296
7	1754.9	98.4149	8	1742.37	98.7569
9	1653.66	100.904	10	1586.16	99.4451
11	1202.4	98.3342	12	1171.54	98.6088
13	1102.12	97.8548	14	1078.98	96.8161

[ピーク検出結果]

No.	位置	強度	No.	位置	強度
15	1055.84	98.7686	16	1024.98	97.6688
17	981.59	98.3764			

Figure 19. The HRESIMS spectrum of **3**.

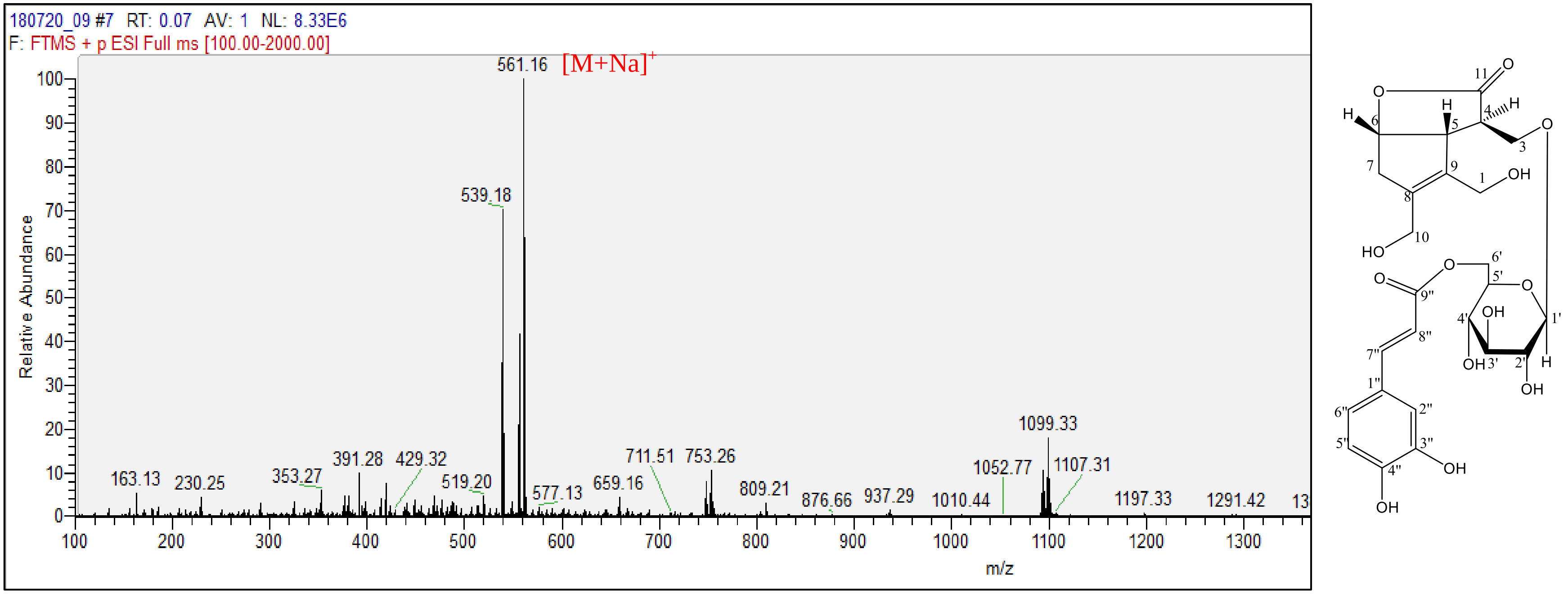

Elemental composition search on mass 561.16

m/z= 556.16-566.16

m/z	Theo. Mass	Delta (mmu)	RDB equiv.	Composition
561.1572	561.1579	-0.63	10.5	$C_{25}H_{30}O_{13}Na$

Figure 20. The ^{1}H NMR spectrum of **3** in CD_3OD, 500 MHz.

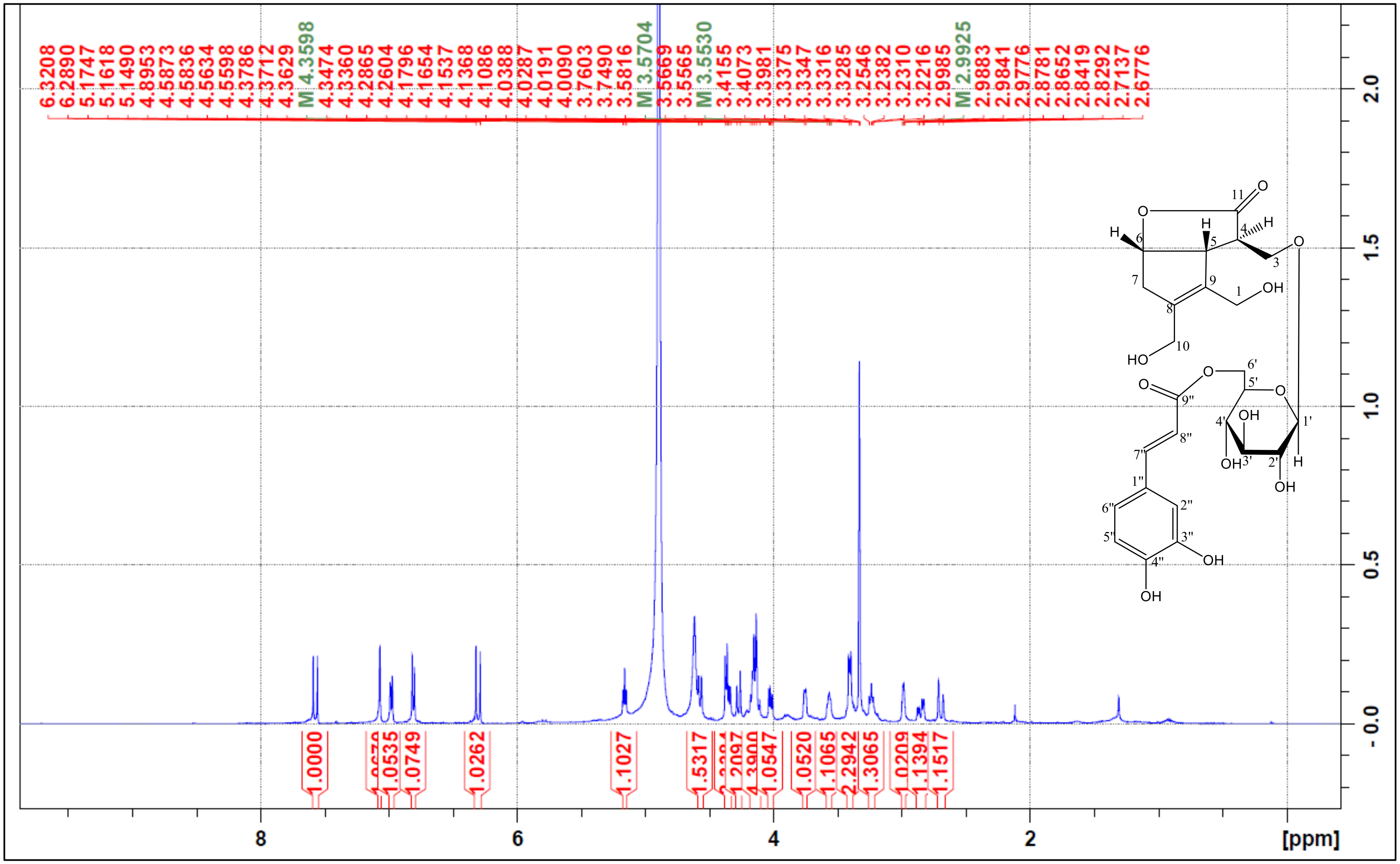

Figure 21. The ^{13}C NMR spectrum of **3** in CD_3OD, 175 MHz.

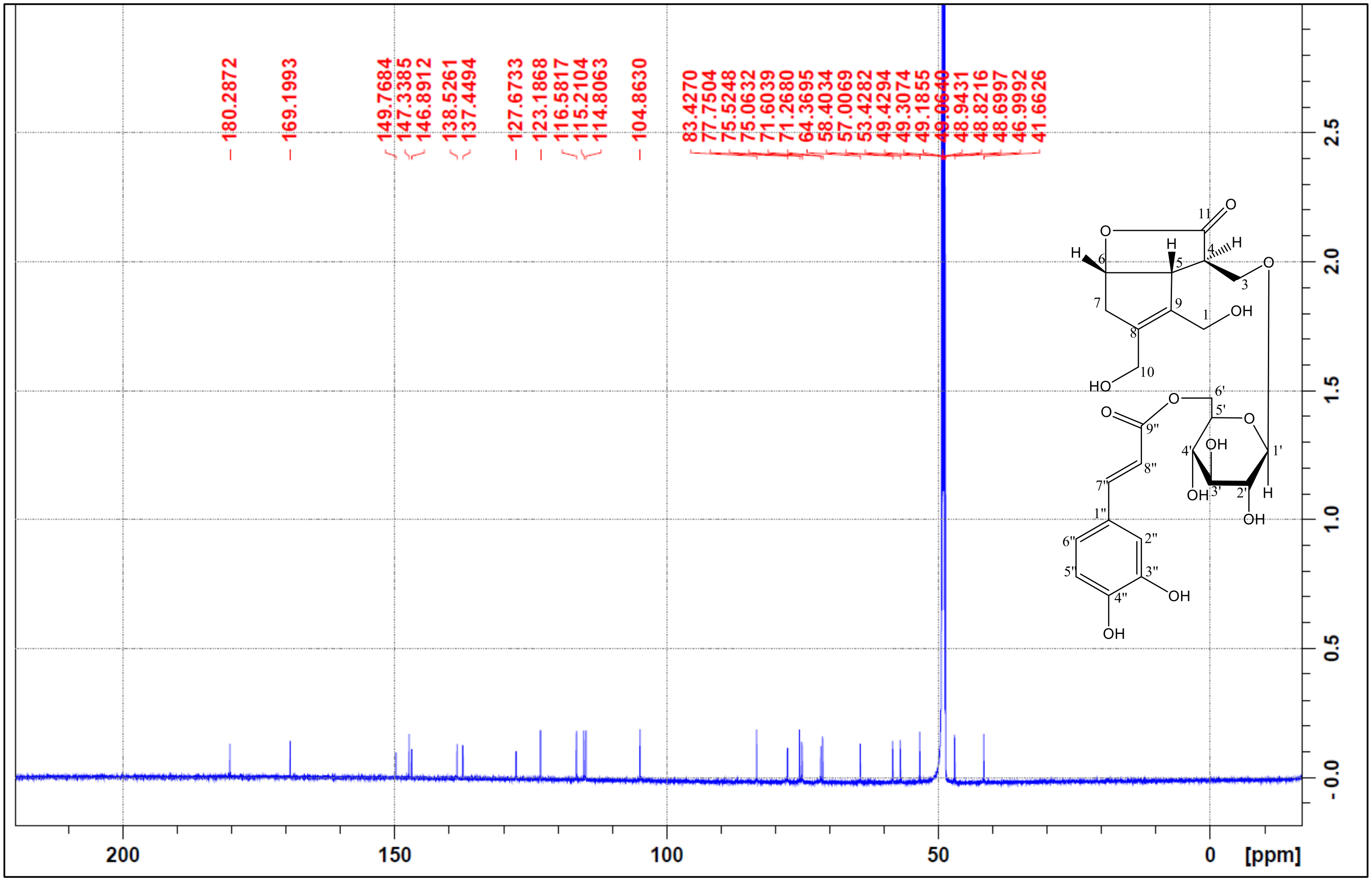

Figure 22. The DEPT-135 NMR spectrum of **3** in CD_3OD, 175 MHz.

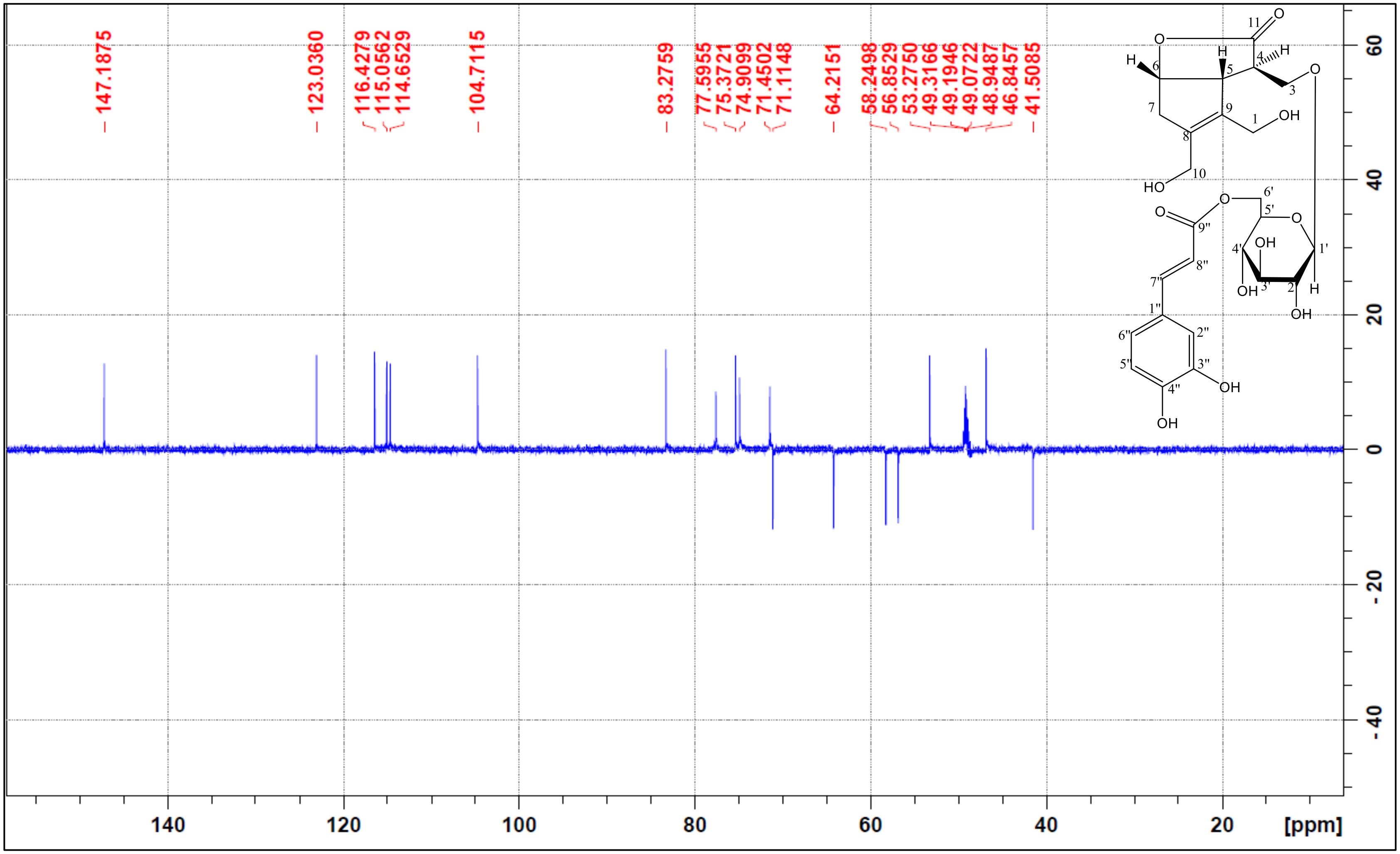

Figure 23. The COSY spectrum of **3** in CD_3OD, 700 MHz.

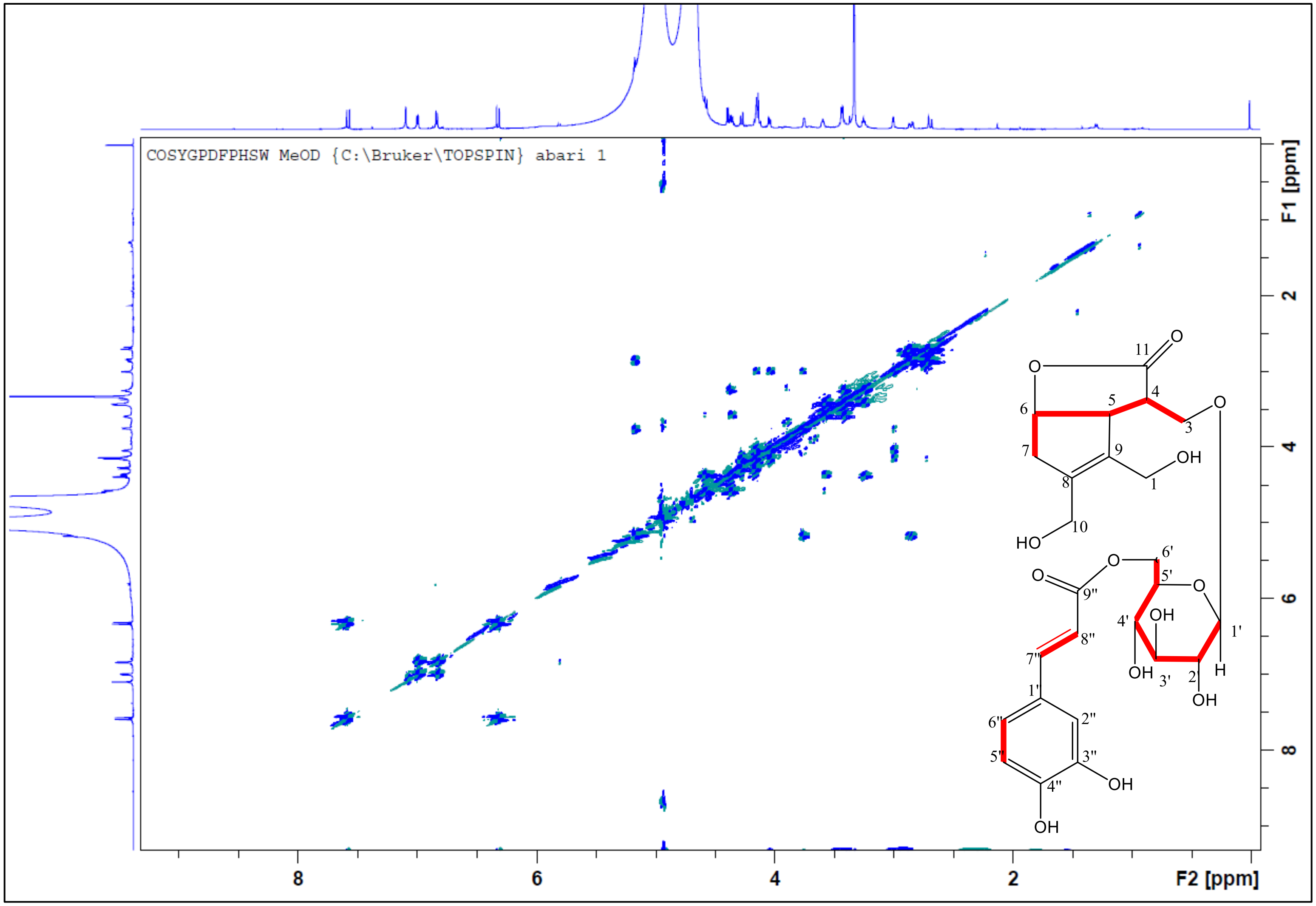

Figure 24. The HSQC spectrum of **3** in CD_3OD, 700 MHz

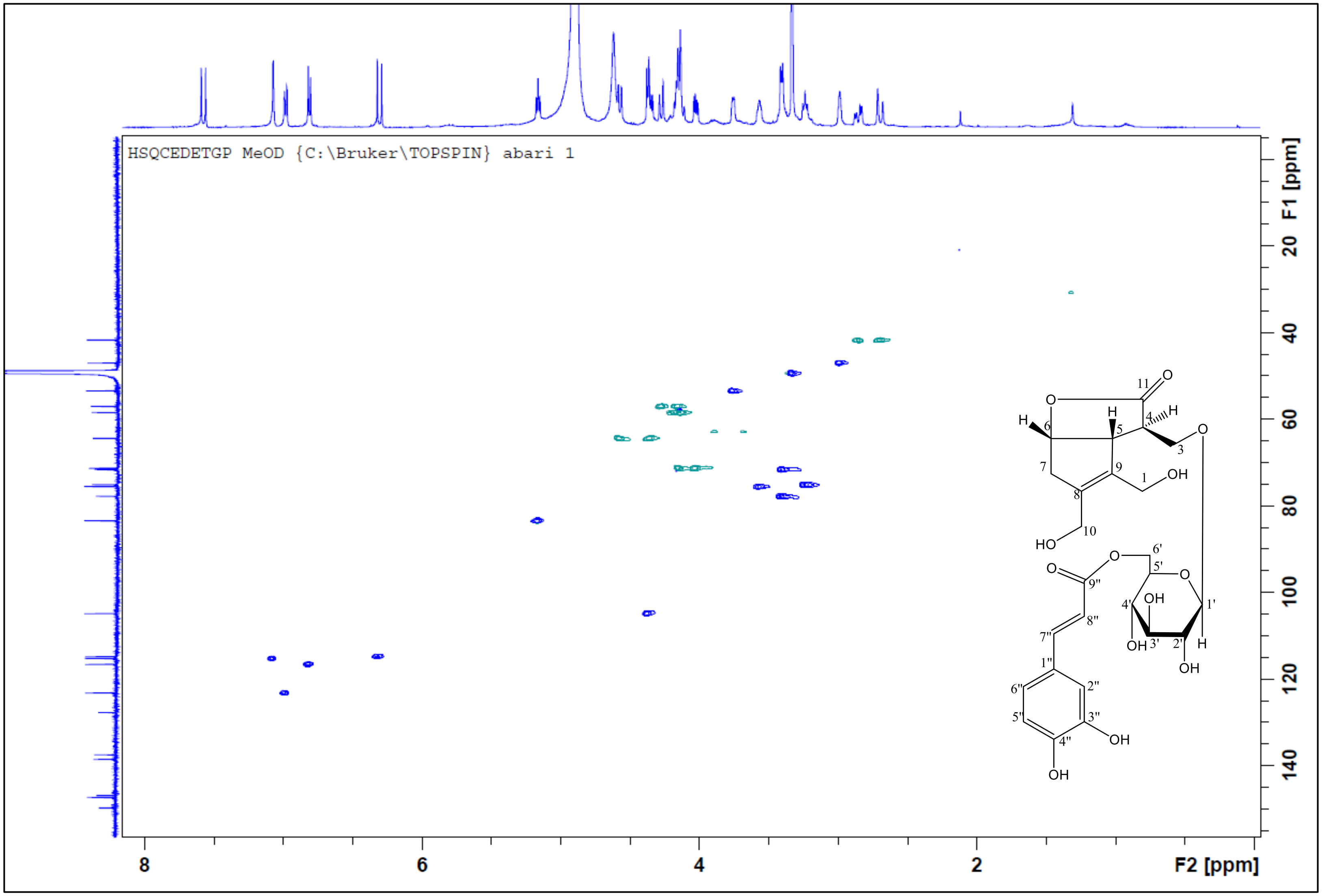

Figure 25. The HSQC spectrum of **3** in CD_3OD, 700 MHz.

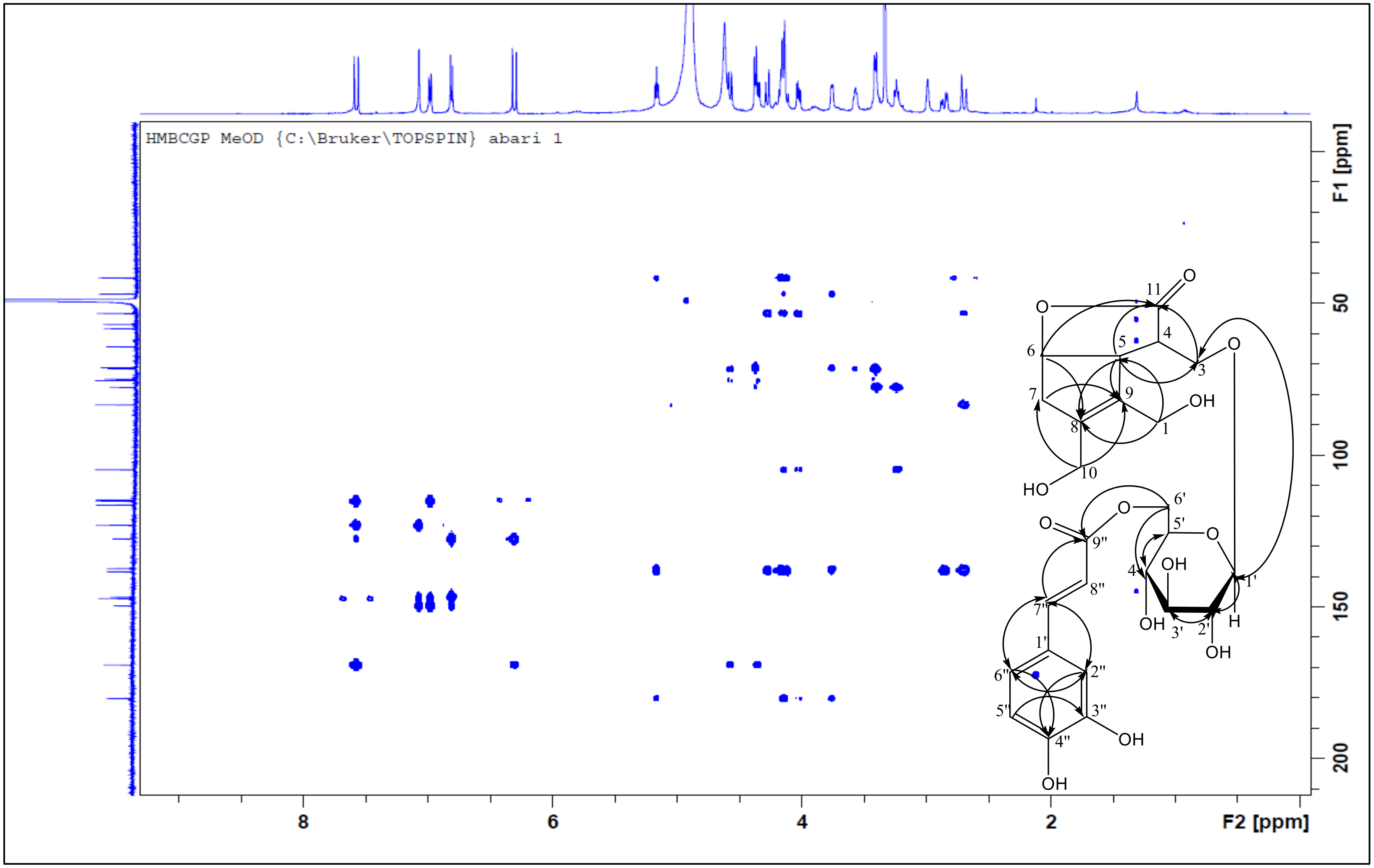

Figure 26. The NOESY spectrum of **2** in CD_3OD, 500 MHz.

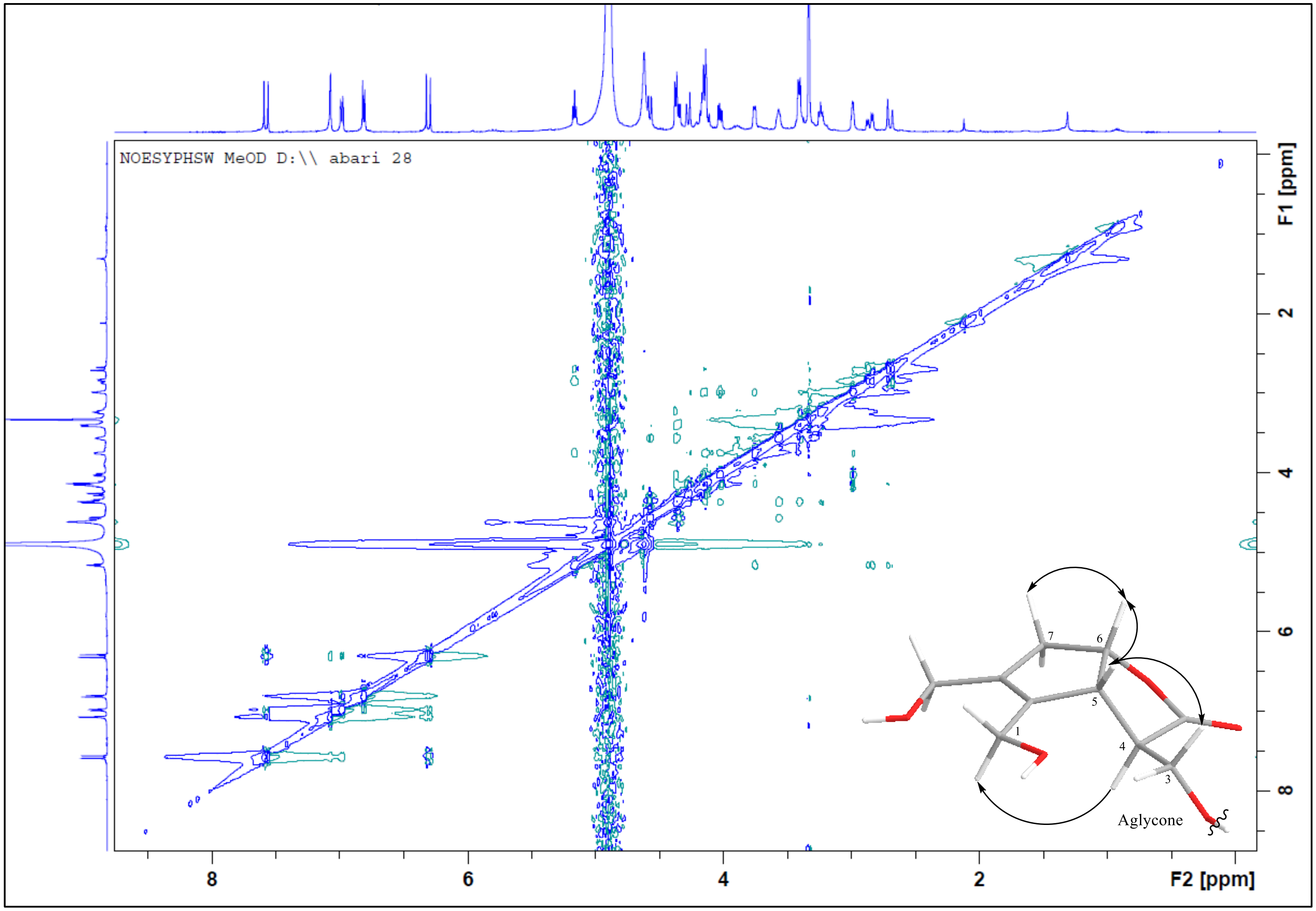

Figure 27. The IR spectrum of **3**.

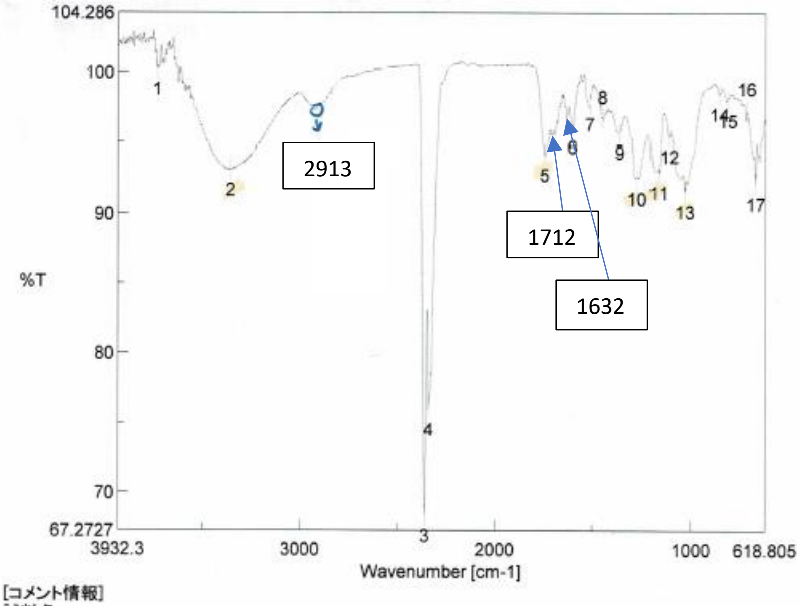

[コメント情報]

試料名	
コメント	
測定者	
所属	
会社	HU

[データ情報]		[測定情報]	
作成日時	2018/06/28 17:04	機種名	FT/IR-4600typeA
		シリアル番号	D015461786
データタイプ	等間隔データ		
横軸	Wavenumber [cm-1]	測定日時	2018/06/28 17:04
縦軸	%T		
スタート	599.753 cm-1	光源	標準光源
エンド	7800.65 cm-1	検出器	TGS
データ間隔	0.964233 cm-1	積算回数	10
データ数	7469	分解	4 cm-1
		ゼロフィリング	On
		アポダイゼーション	Cosine
		ゲイン	Auto (2)
		アパーチャー	Auto (7.1 mm)
		スキャンスピード	Auto (2 mm/sec)
		フィルタ	Auto (30000 Hz)

[ピーク検出結果]

No.	位置	強度	No.	位置	強度
1	3732.55	100.201	2	3356.5	93.0717
3	2361.41	68.3232	4	2341.16	75.9363
5	1745.26	94.149	6	1600.63	96.1241
7	1512.88	97.7611	8	1448.28	96.8026
9	1360.53	95.7487	10	1274.72	92.4856
11	1165.76	92.9243	12	1111.76	95.487
13	1032.69	91.5669	14	856.239	98.461

[ピーク検出結果]

No.	位置	強度	No.	位置	強度
15	813.813	98.0127	16	714.497	97.4101
17	669.178	92.1315			

Figure 28. The UV spectrum of **3**.

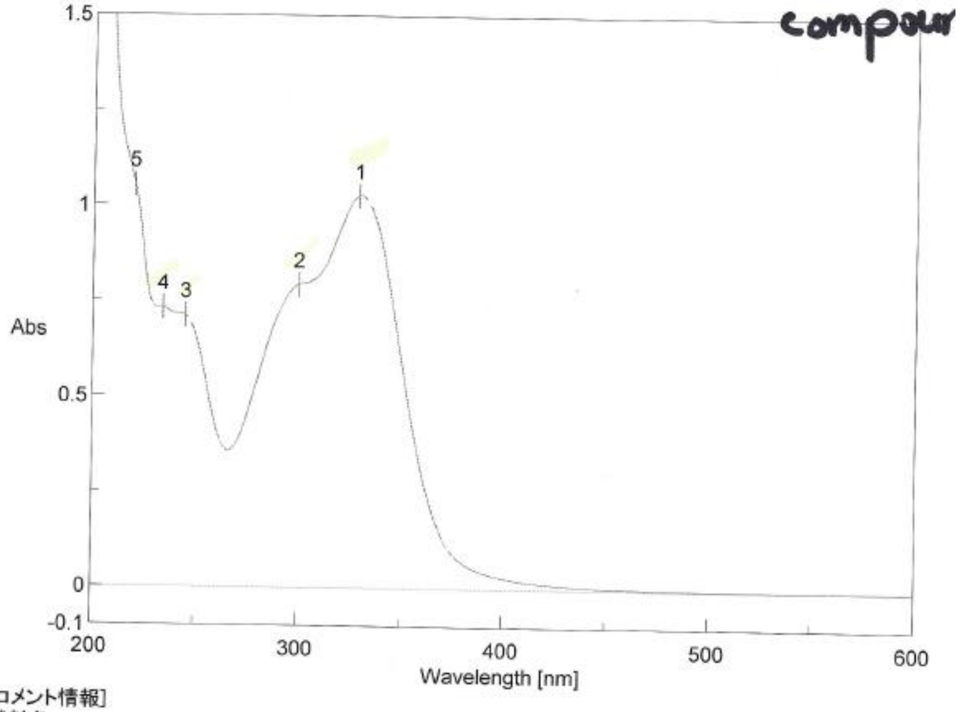

[コメント情報]

試料名	
コメント	
測定者	
所属	
会社	HU

[データ情報]

作成日時	2018/07/02 20:30
データタイプ	等間隔データ
横軸	Wavelength [nm]
縦軸	Abs
スタート	600 nm
エンド	200 nm
データ間隔	1 nm
データ数	401

[測定情報]

装置名	V-630
機種名	V-630
シリアル番号	A042161148
測定日時	2018/07/02 20:29
測光モード	Abs
測定範囲	600 - 200 nm
データ取込間隔	1 nm
UV/Vis バンド幅	1.5 nm
レスポンス	Fast
走査速度	400 nm/min
光源切換	340 nm
光源	D2/WI
フィルタ切換	ステップ
補正	ベースライン

[ピーク検出結果]

No.	位置	強度	No.	位置	強度
1	329	1.02845	2	300	0.795039
3	245	0.711464	4	234	0.732239
5	220	1.05375			

Figure 29. The HRESIMS spectrum of **4**.

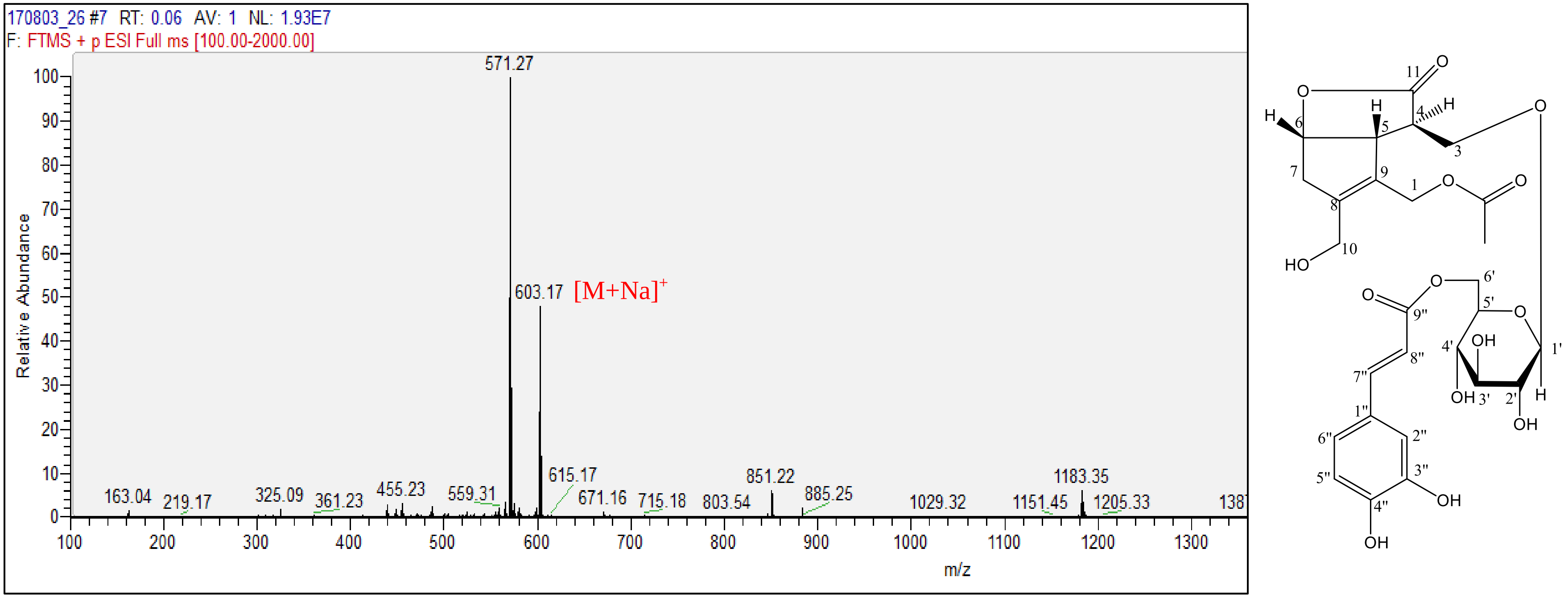

Elemental composition search on mass 603.17

m/z= 598.17-608.17

m/z	Theo. Mass	Delta (mmu)	RDB equiv.	Composition
603.1685	603.1684	0.03	11.5	$C_{27}H_{32}O_{14}Na$

Figure 30. The ^{1}H NMR spectrum of **4** in CD_3OD, 500 MHz.

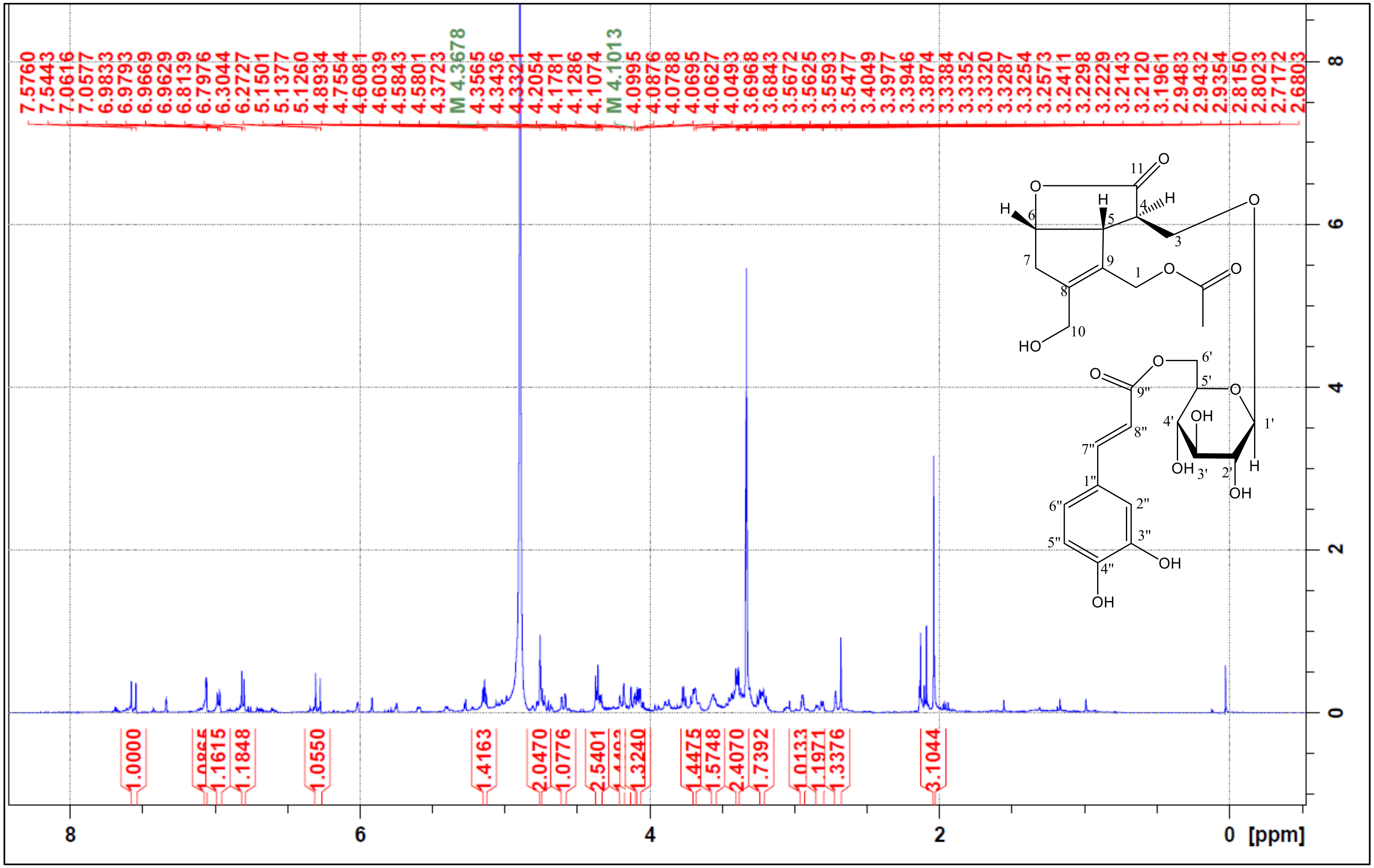

Figure 31. The ^{13}C NMR spectrum of **3** in CD_3OD, 175 MHz.

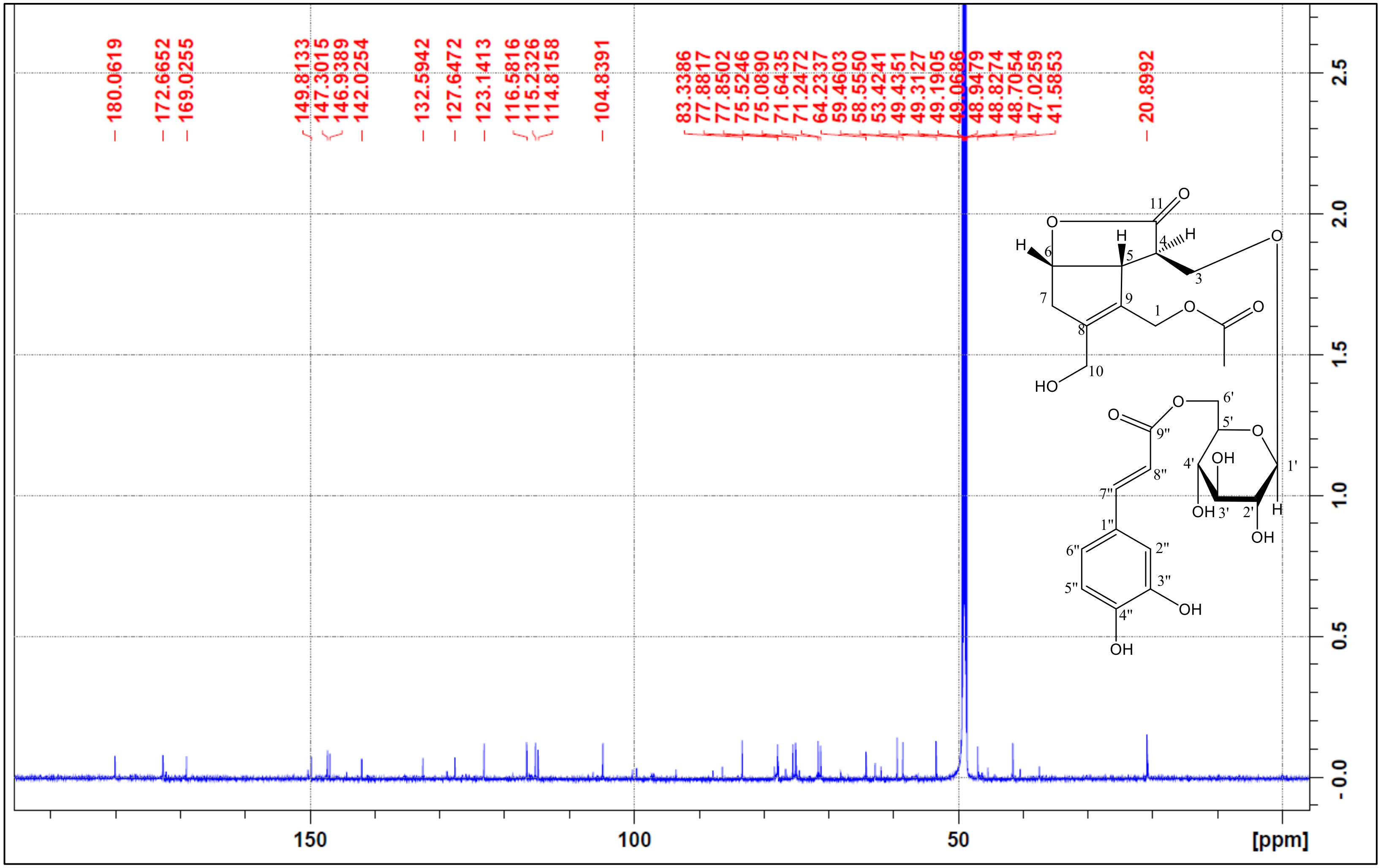

Figure 32. The DEPT-135 NMR spectrum of **4** in CD_3OD, 175 MHz.

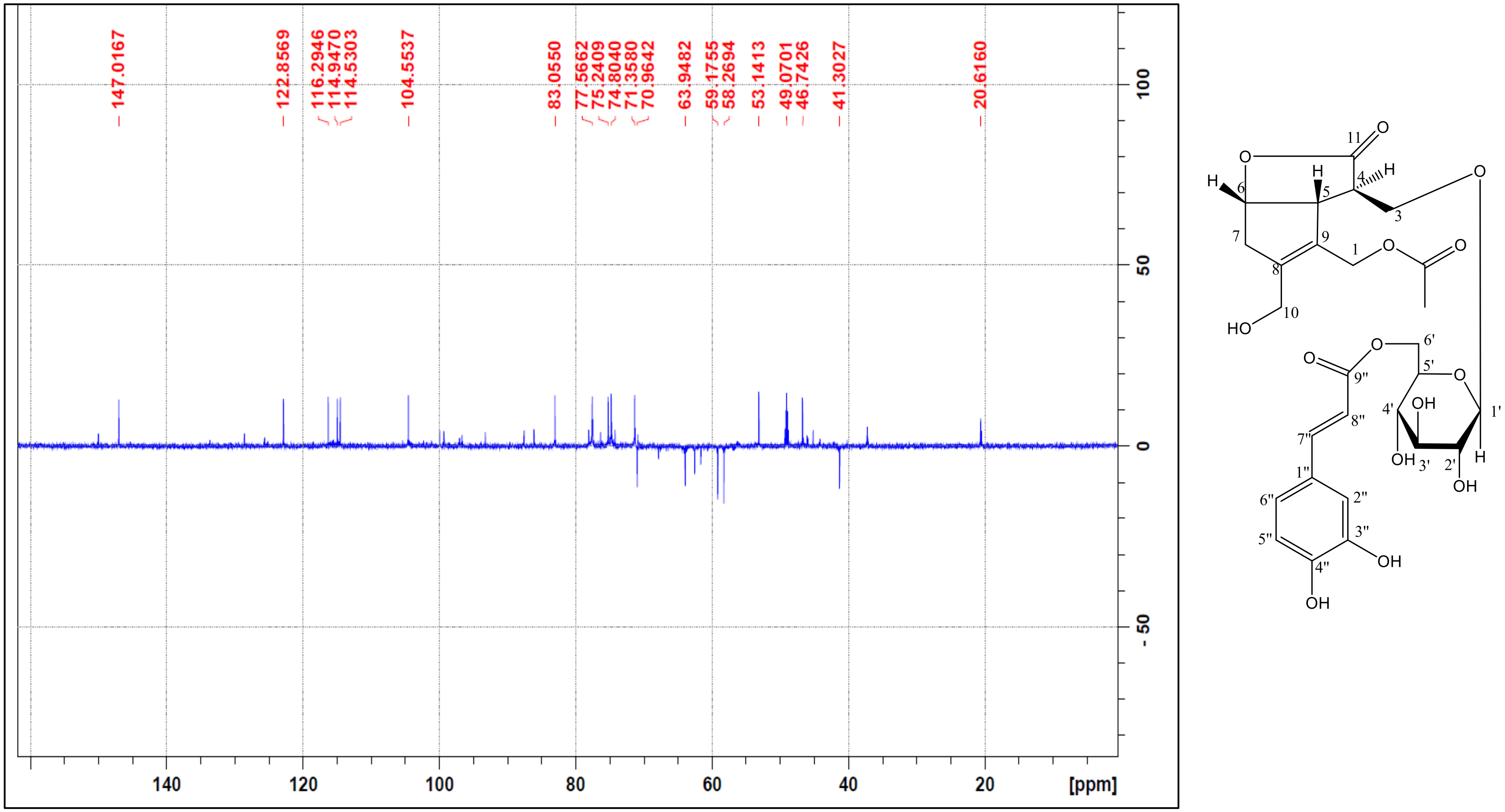

Figure 33. The COSY spectrum of **4** in CD_3OD, 500 MHz.

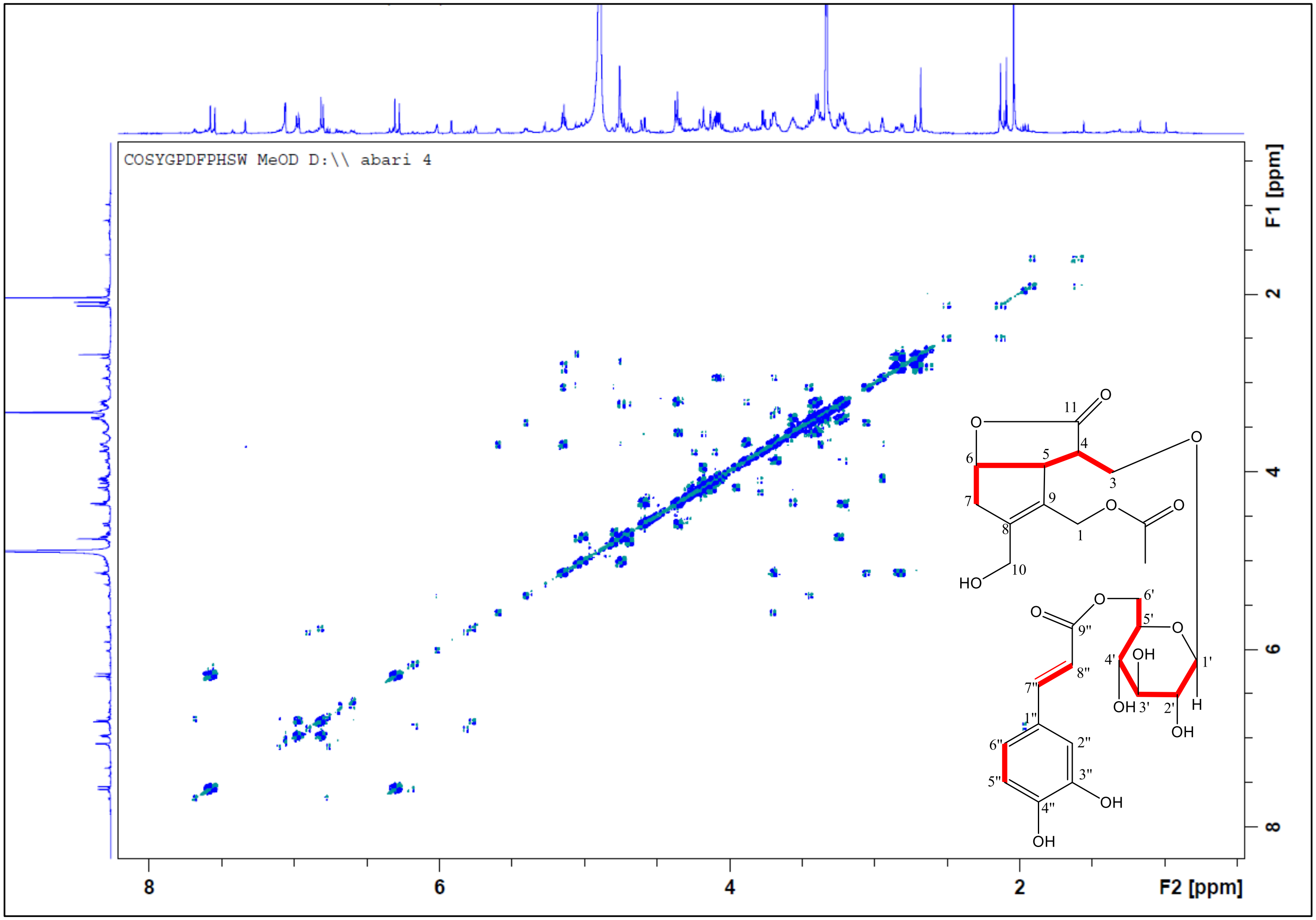

Figure 34. The HSQC spectrum of **4** in CD_3OD, 700 MHz.

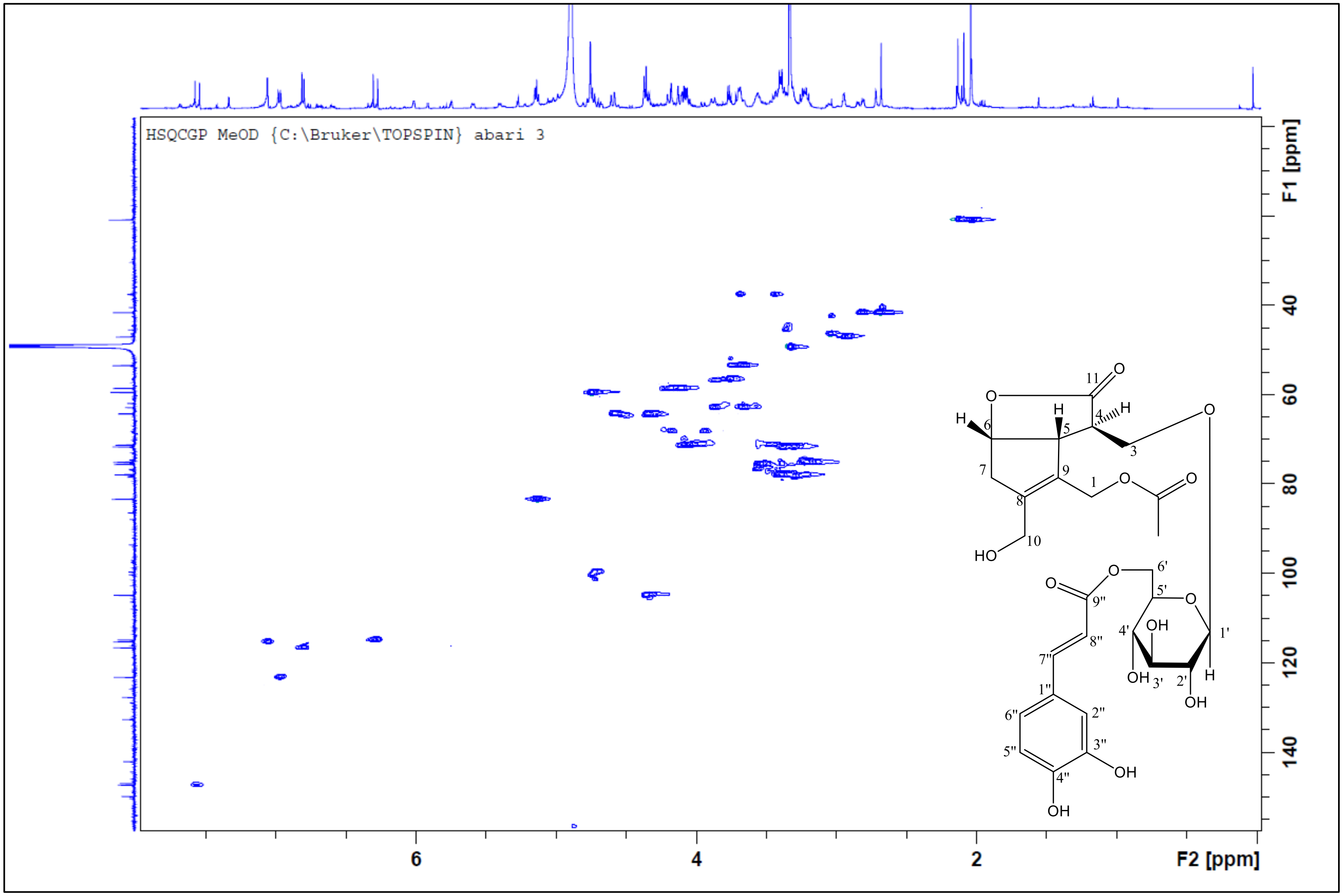

Figure 35. The HMBC spectrum of **4** in CD_3OD, 700 MHz.

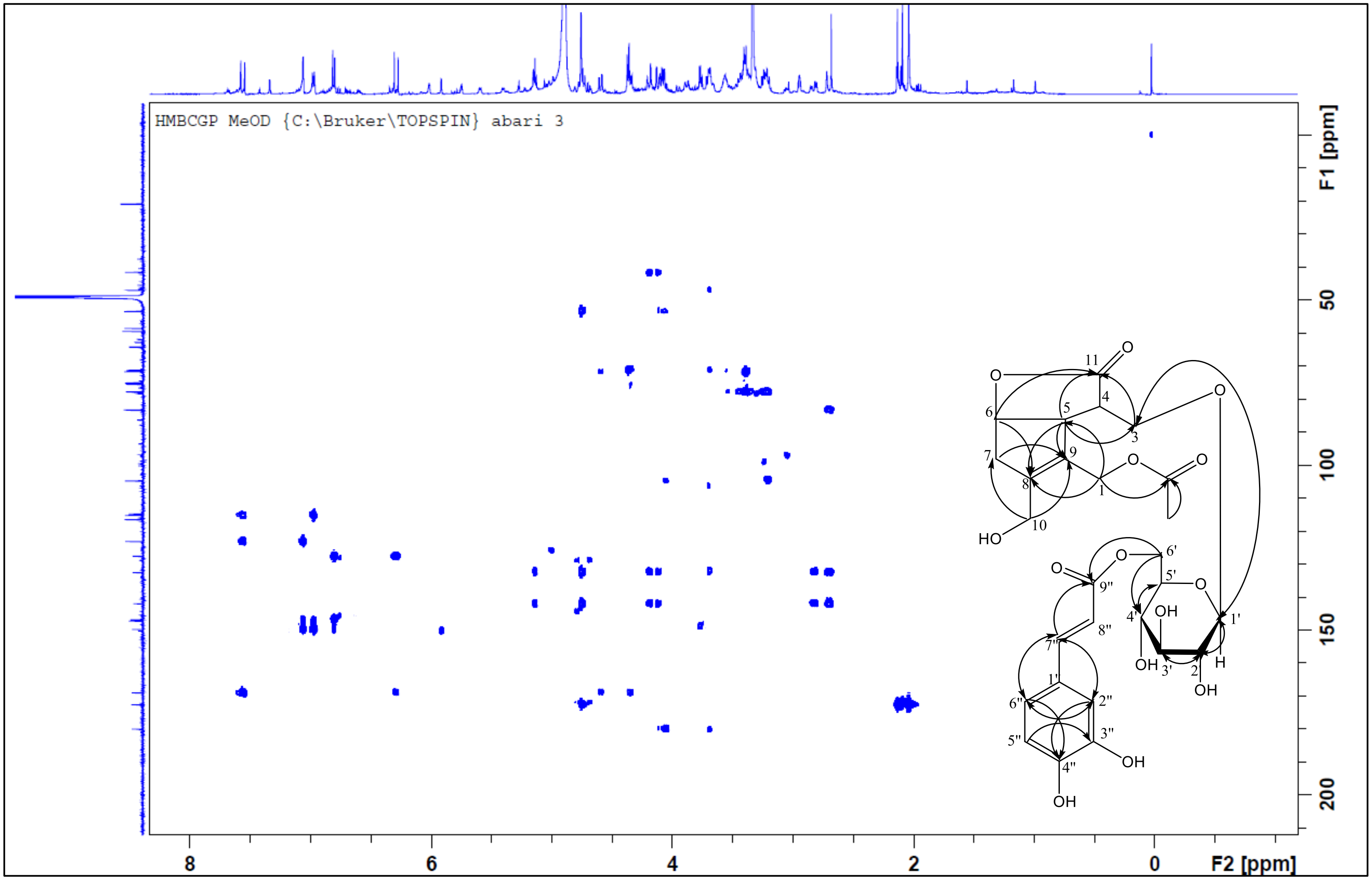

Figure 36. The NOESY spectrum of **4** in CD_3OD, 500 MHz.

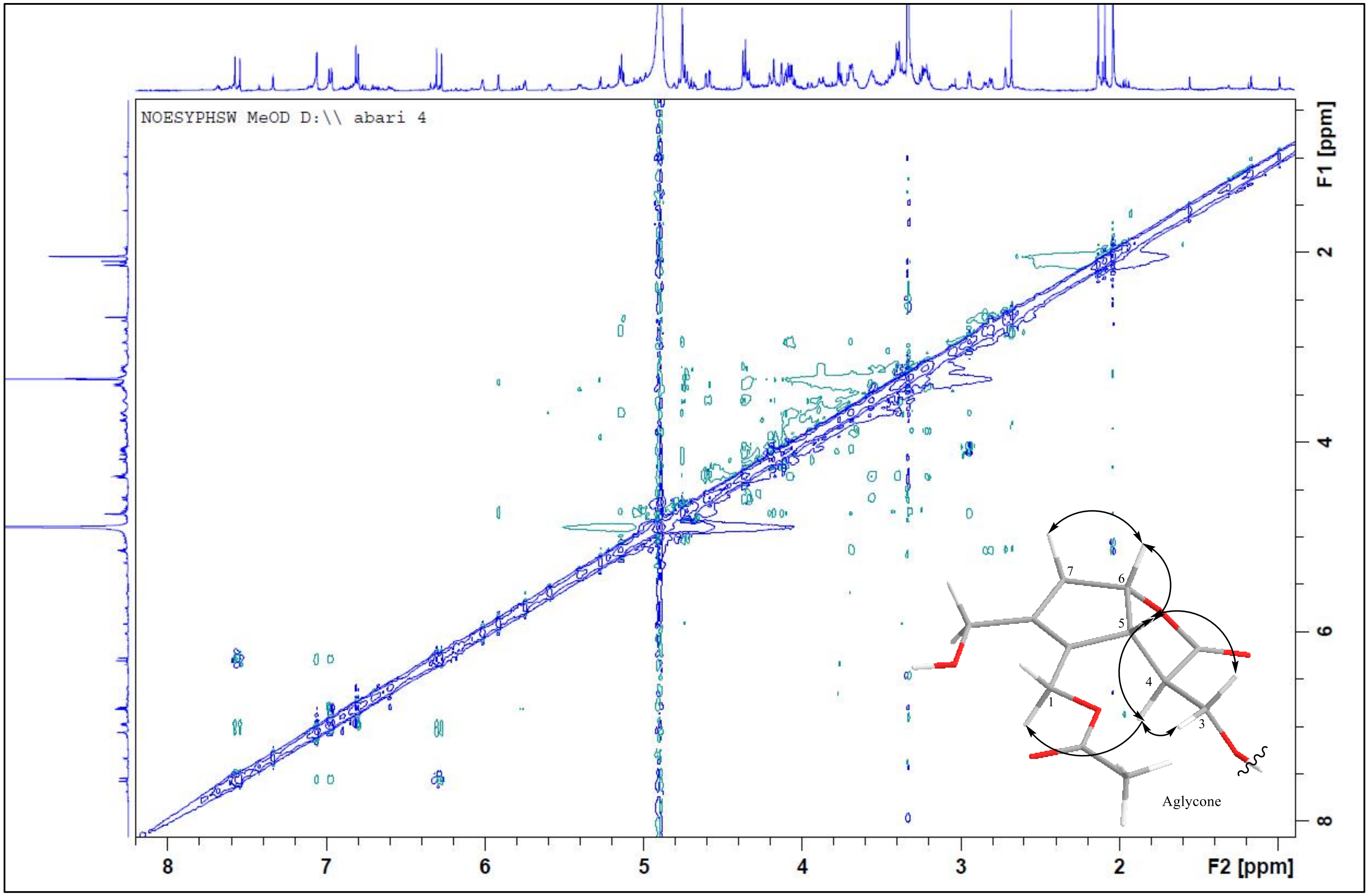

Figure 37. The IR spectrum of **4**.

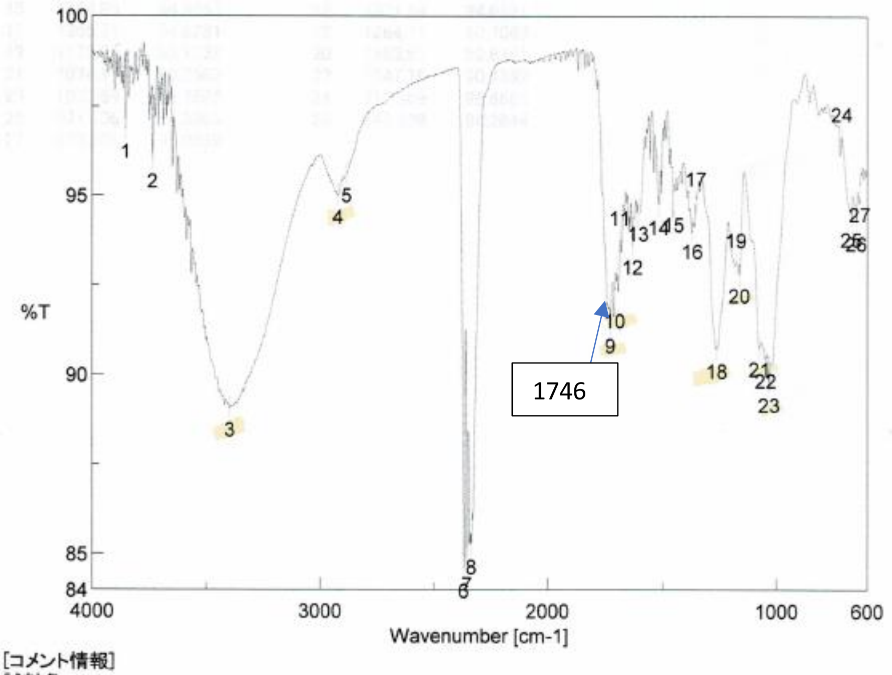

[コメント情報]
試料名
コメント
測定者
所属
会社 HU

[データ情報]
作成日時 2018/06/28 16:34
データタイプ 等間隔データ
横軸 Wavenumber [cm-1]
縦軸 %T
スタート 599.753 cm-1
エンド 7800.65 cm-1
データ間隔 0.964233 cm-1
データ数 7469

[測定情報]
機種名 FT/IR-4600typeA
シリアル番号 D015461786
測定日時 2018/06/28 16:34
光源 標準光源
検出器 TGS
積算回数 10
分解 4 cm-1
ゼロフィリング On
アポダイゼーション Cosine
ゲイン Auto (2)
アパーチャー Auto (7.1 mm)
スキャンスピード Auto (2 mm/sec)
フィルタ Auto (30000 Hz)

[ピーク検出結果]

No.	位置	強度	No.	位置	強度
1	3852.11	96.8495	2	3733.51	96.0049
3	3396.03	89.0751	4	2920.66	95.0033
5	2883.06	95.5993	6	2367.19	84.5837
7	2354.66	84.7644	8	2335.37	85.245
9	1732.73	91.4031	10	1713.44	92.1058
11	1690.3	93.7358	12	1634.38	93.6187
13	1608.34	94.5546	14	1518.67	94.7232

[ピーク検出結果]

No.	位置	強度	No.	位置	強度
15	1454.06	94.8053	16	1371.14	94.0681
17	1355.71	94.8281	18	1264.11	90.7063
19	1179.26	93.1239	20	1163.83	92.8161
21	1074.16	90.7582	22	1047.16	90.4392
23	1032.69	89.7685	24	712.569	96.6683
25	671.106	94.3969	26	648.929	94.2844
27	633.501	95.0969			

Figure 38. The UV spectrum of **4**.

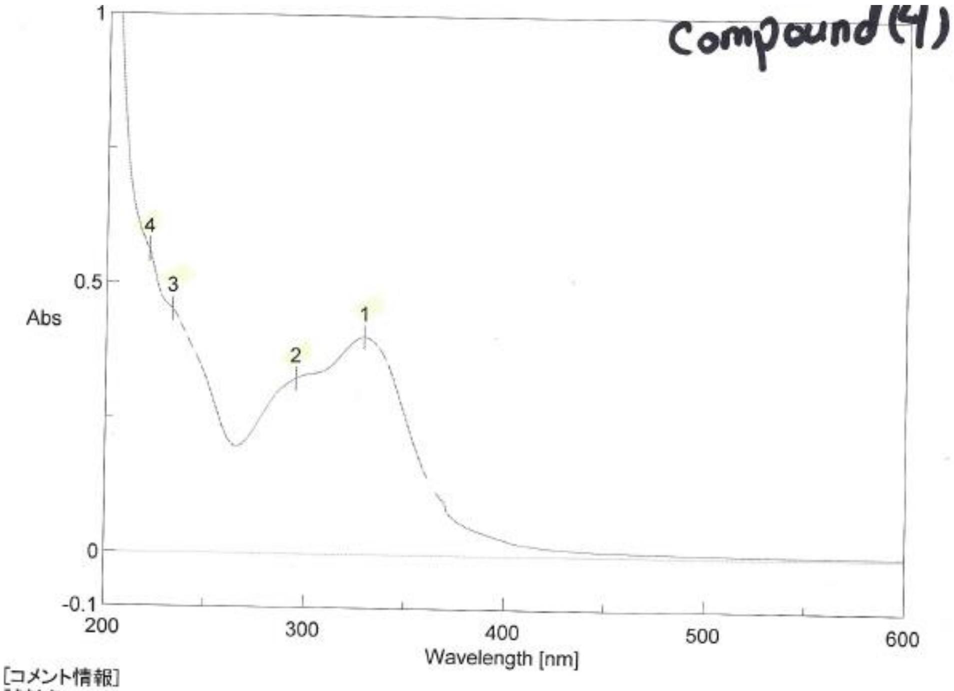

[コメント情報]

試料名	
コメント	
測定者	
所属	
会社	HU

[データ情報]

作成日時	2018/07/02 19:44
データタイプ	等間隔データ
横軸	Wavelength [nm]
縦軸	Abs
スタート	600 nm
エンド	200 nm
データ間隔	1 nm
データ数	401

[測定情報]

装置名	V-630
機種名	V-630
シリアル番号	A042161148
測定日時	2018/07/02 19:42
測光モード	Abs
測定範囲	600 - 200 nm
データ取込間隔	1 nm
UV/Vis バンド幅	1.5 nm
レスポンス	Fast
走査速度	400 nm/min
光源切換	340 nm
光源	D2/WI
フィルタ切換	ステップ
補正	ベースライン

[ピーク検出結果]

No.	位置	強度	No.	位置	強度
1	329	0.403842	2	295	0.325442
3	233	0.453021	4	221	0.561628

Figure 39. The HRESIMS spectrum of **5**.

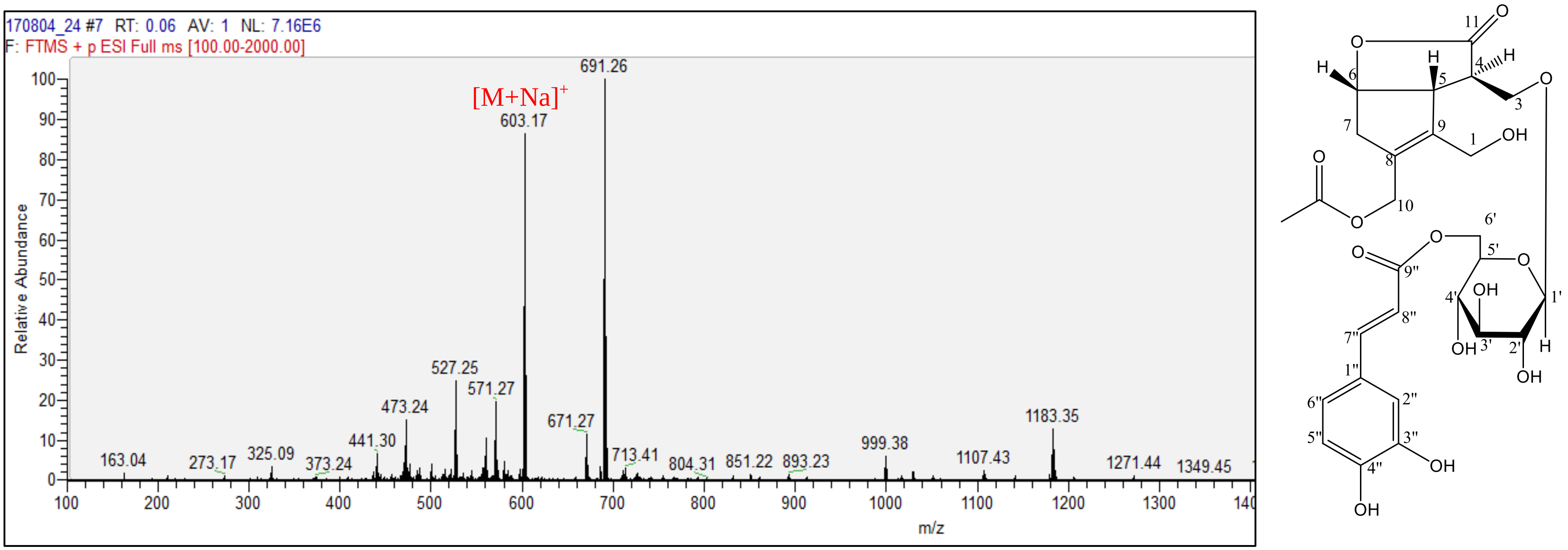

Elemental composition search on mass 603.17

m/z= 598.17-608.17

m/z	Theo. Mass	Delta (mmu)	RDB equiv.	Composition
603.1682	603.1684	-0.22	11.5	$C_{27}H_{32}O_{14}Na$

Figure 40. The ^{1}H NMR spectrum of **5** in CD_3OD, 500 MHz

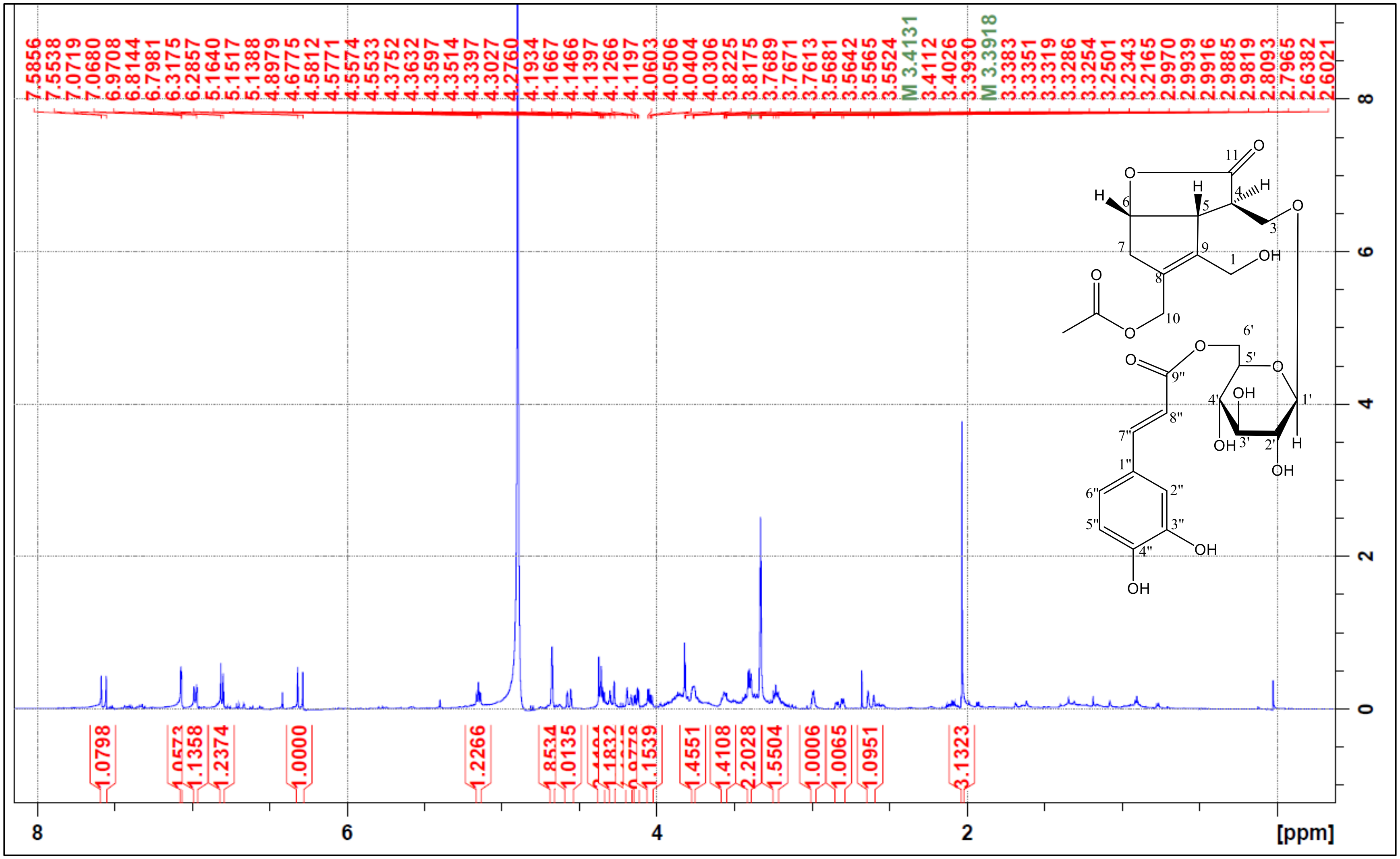

Figure 41. The ^{13}C NMR spectrum of **5** in CD_3OD, 175 MHz.

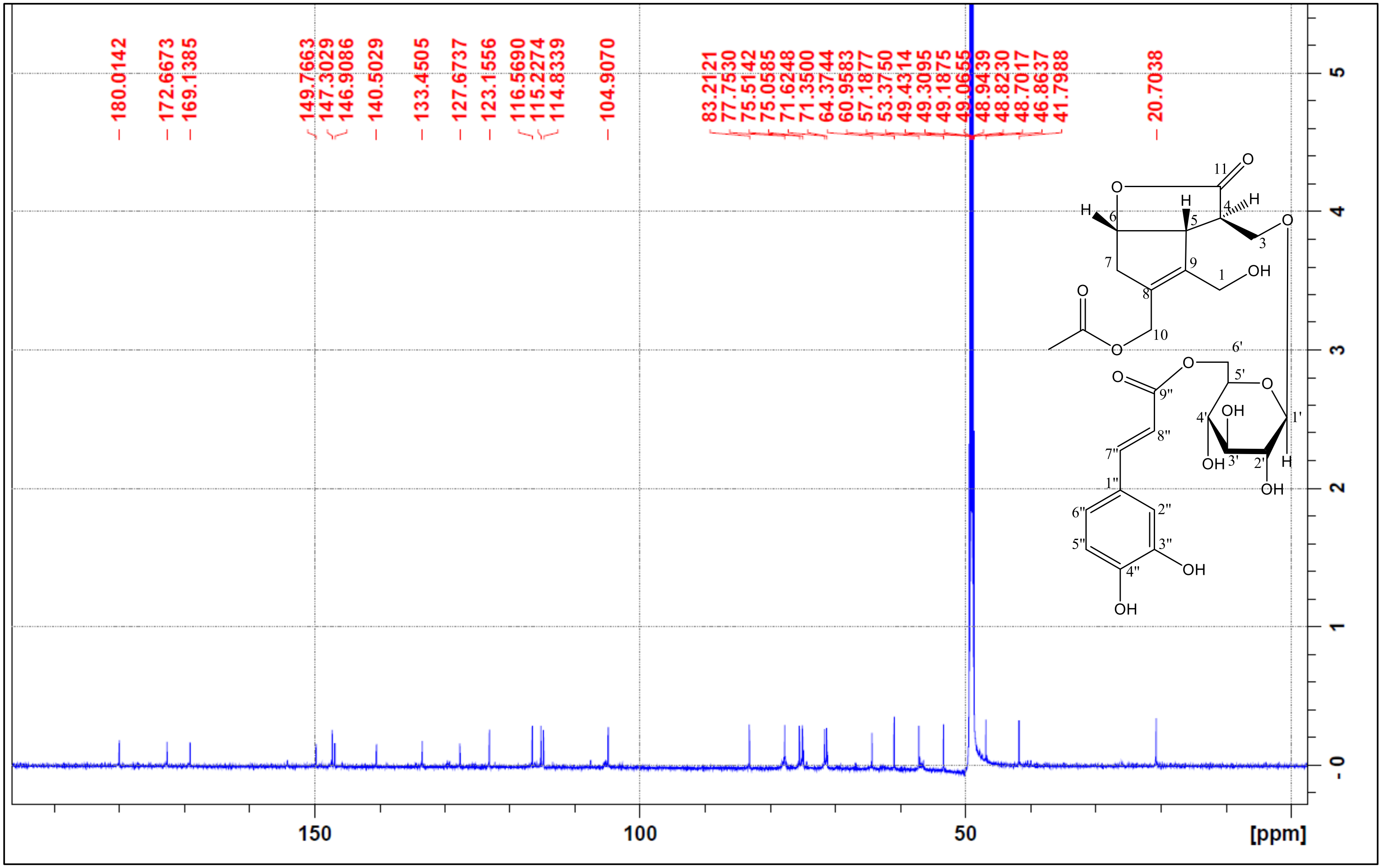

Figure 42. The DEPT-135 NMR spectrum of **5** in CD_3OD, 175 MHz.

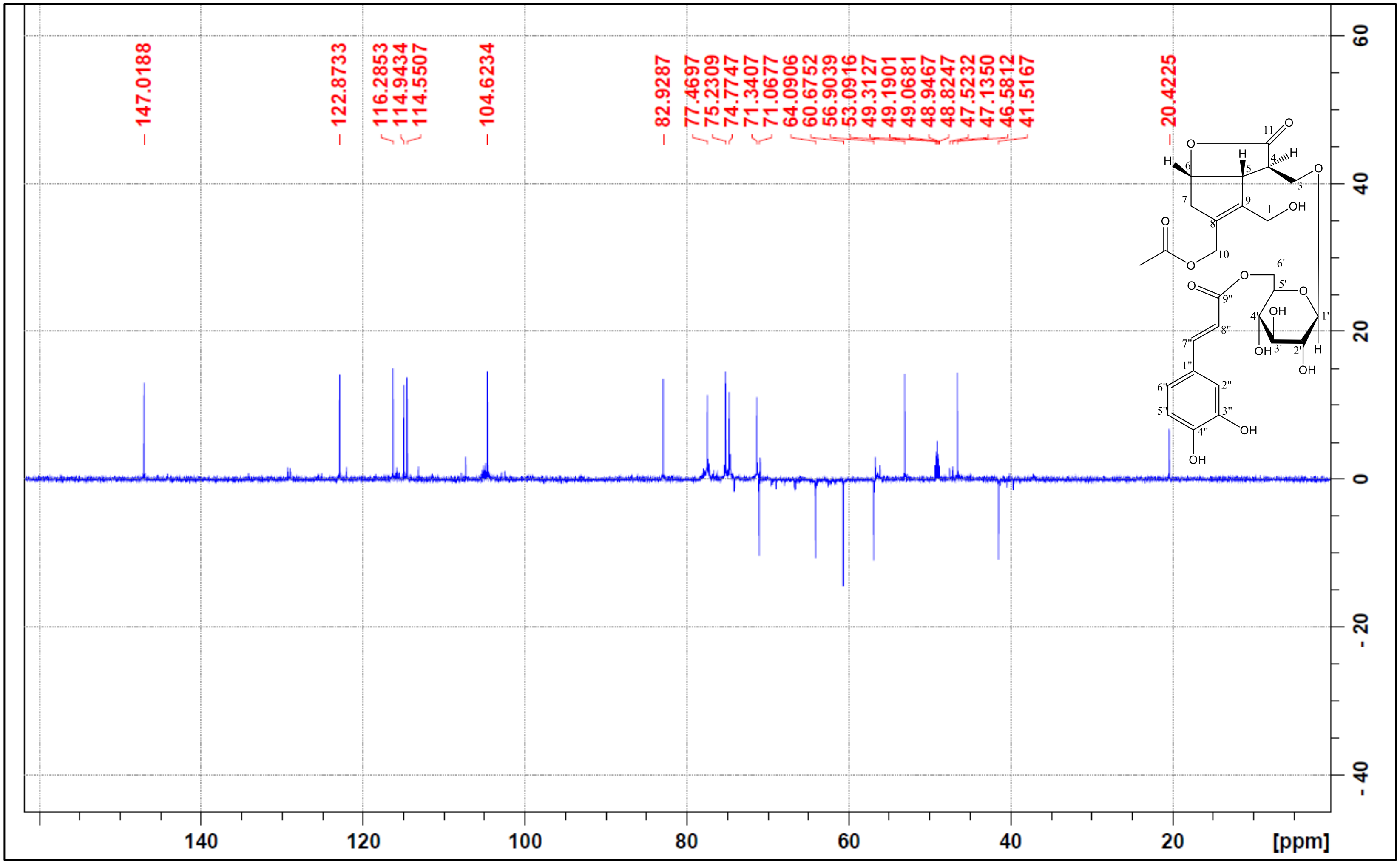

Figure 43. The COSY spectrum of **5** in CD_3OD, 500 MHz.

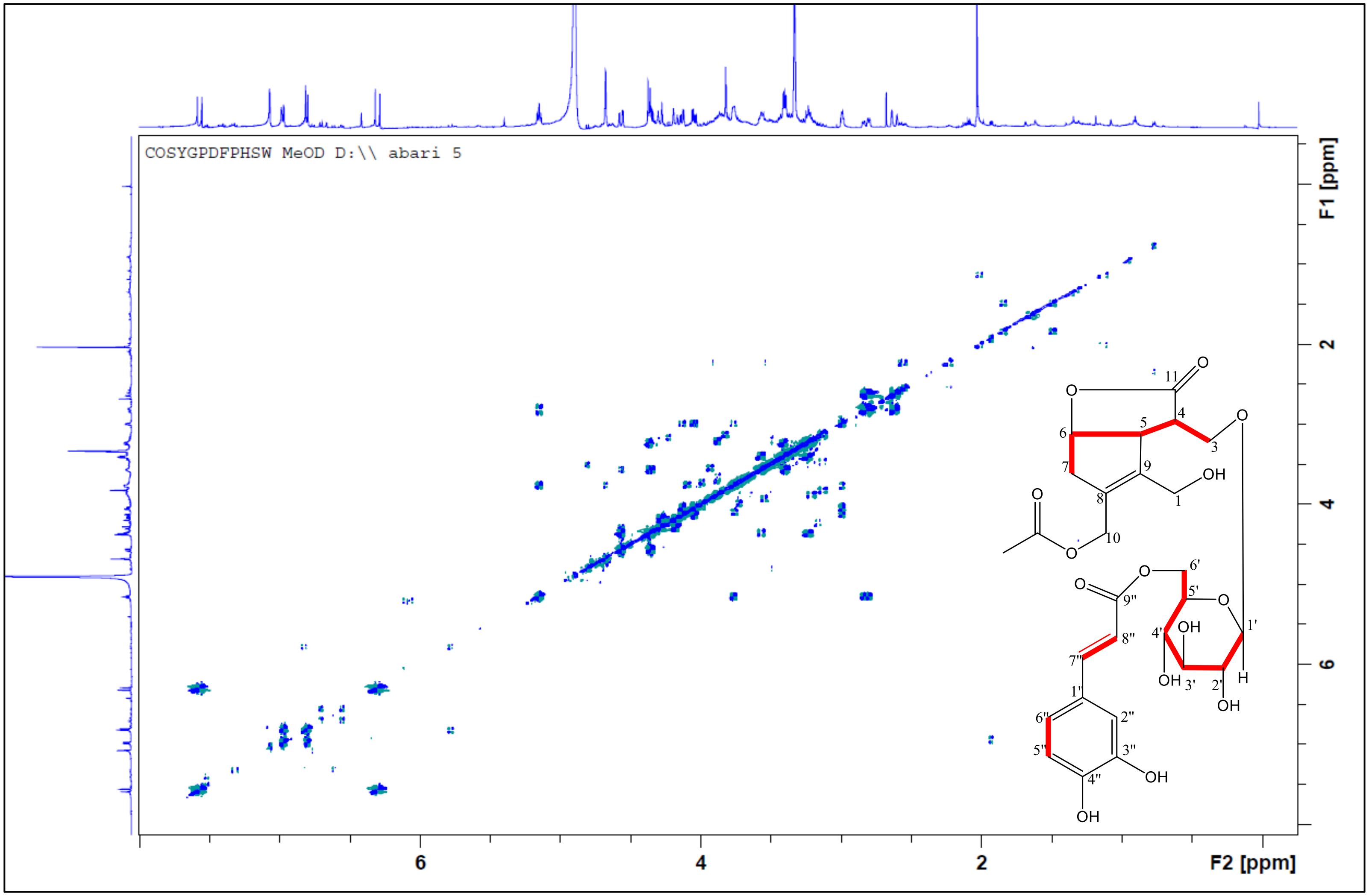

Figure 44. The HSQC spectrum of **5** in CD_3OD, 700 MHz.

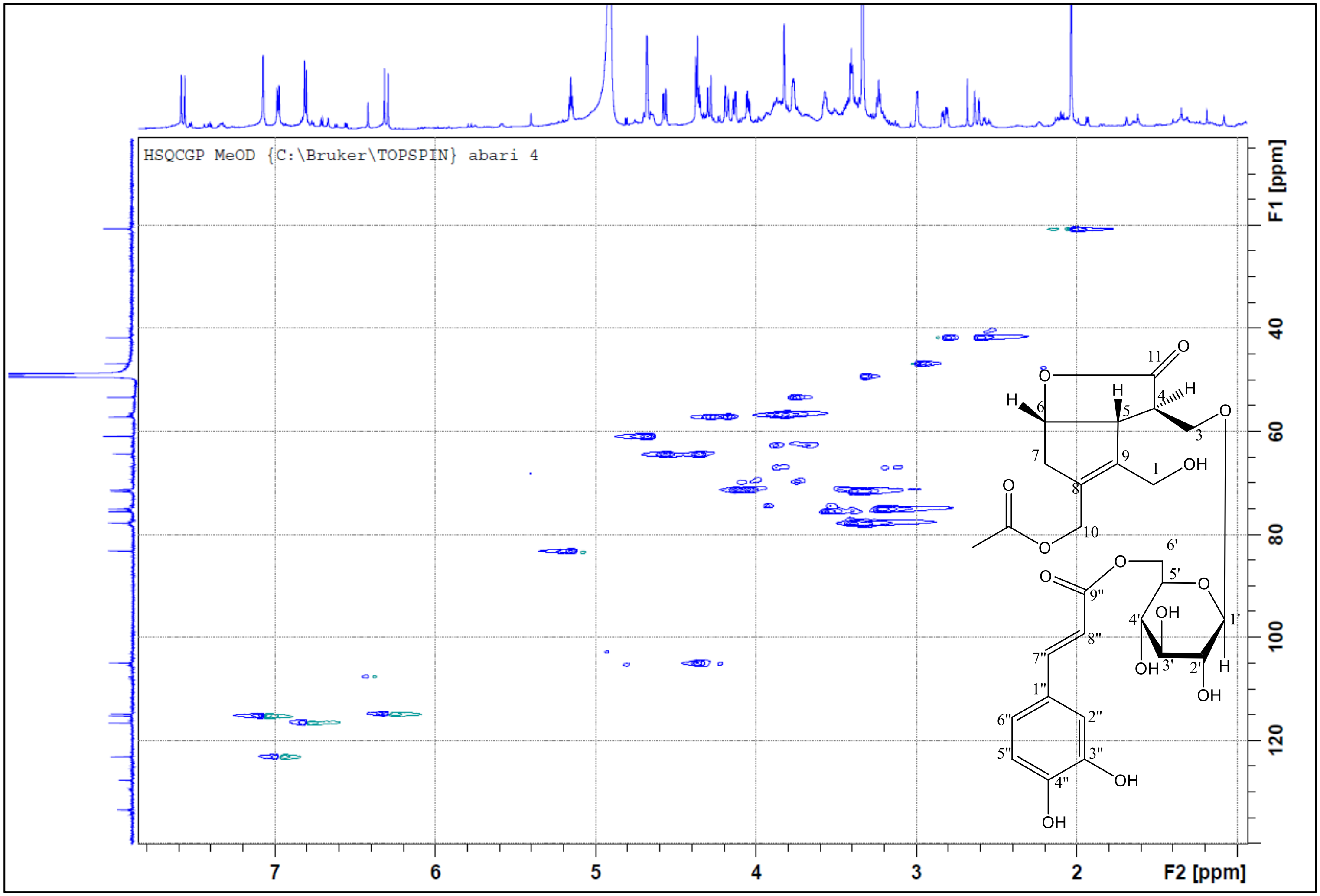

Figure 45. The HMBC spectrum of **5** in CD_3OD, 700 MHz.

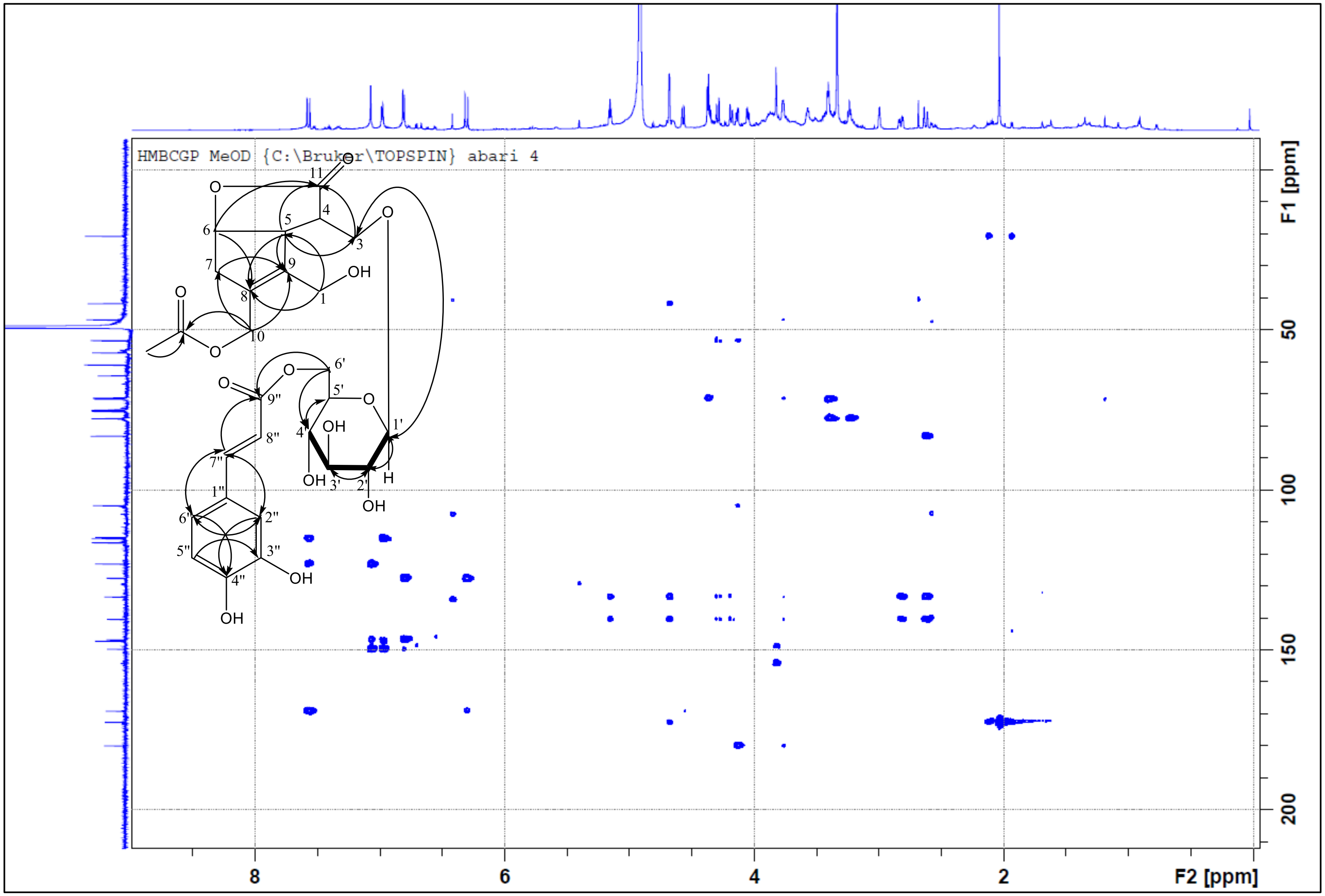

Figure 46. The NOESY spectrum of **5** in CD_3OD, 500 MHz.

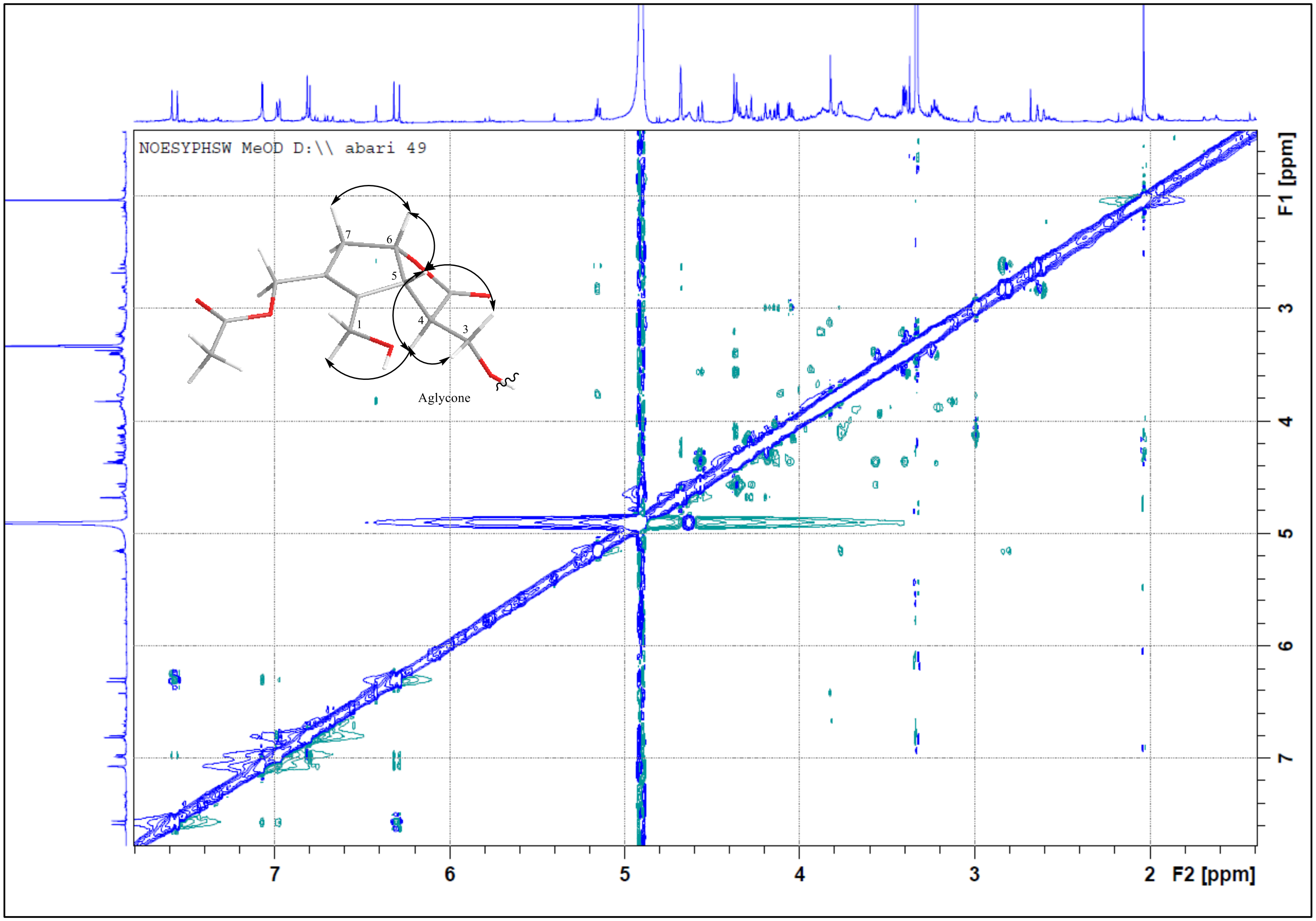

Figure 47. The IR spectrum of **5**.

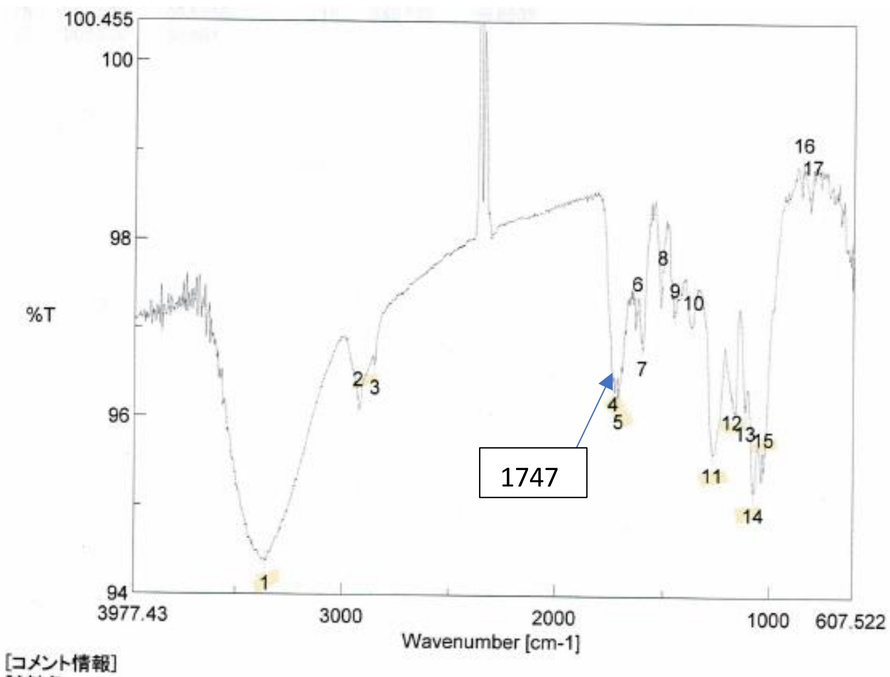

[コメント情報]
試料名
コメント
測定者
所属
会社　HU

[データ情報]

作成日時	2018/07/04 16:03
データタイプ	等間隔データ
横軸	Wavenumber [cm-1]
縦軸	%T
スタート	599.753 cm-1
エンド	7800.65 cm-1
データ間隔	0.964233 cm-1
データ数	7469

[測定情報]

機種名	FT/IR-4600typeA
シリアル番号	D015461786
測定日時	2018/07/04 16:03
光源	標準光源
検出器	TGS
積算回数	10
分解	4 cm-1
ゼロフィリング	On
アポダイゼーション	Cosine
ゲイン	Auto (2)
アパーチャー	Auto (7.1 mm)
スキャンスピード	Auto (2 mm/sec)
フィルタ	Auto (30000 Hz)

[ピーク検出結果]

No.	位置	強度	No.	位置	強度
1	3361.32	94.3708	2	2928.38	96.1635
X 3	2850.27	96.5796	4	1738.51	96.4149
5	1714.41	96.2203	6	1625.7	97.2733
7	1602.56	96.8155	8	1509.03	97.576
9	1451.17	97.2008	10	1365.35	97.0659
11	1271.82	95.6231	12	1179.26	96.2215
13	1116.58	96.1	14	1078.01	95.1741

[ピーク検出結果]

No.	位置	強度	No.	位置	強度
15	1030.77	95.5256	16	848.525	98.8539
17	806.099	98.601			

Figure 48. The UV spectrum of **5**.

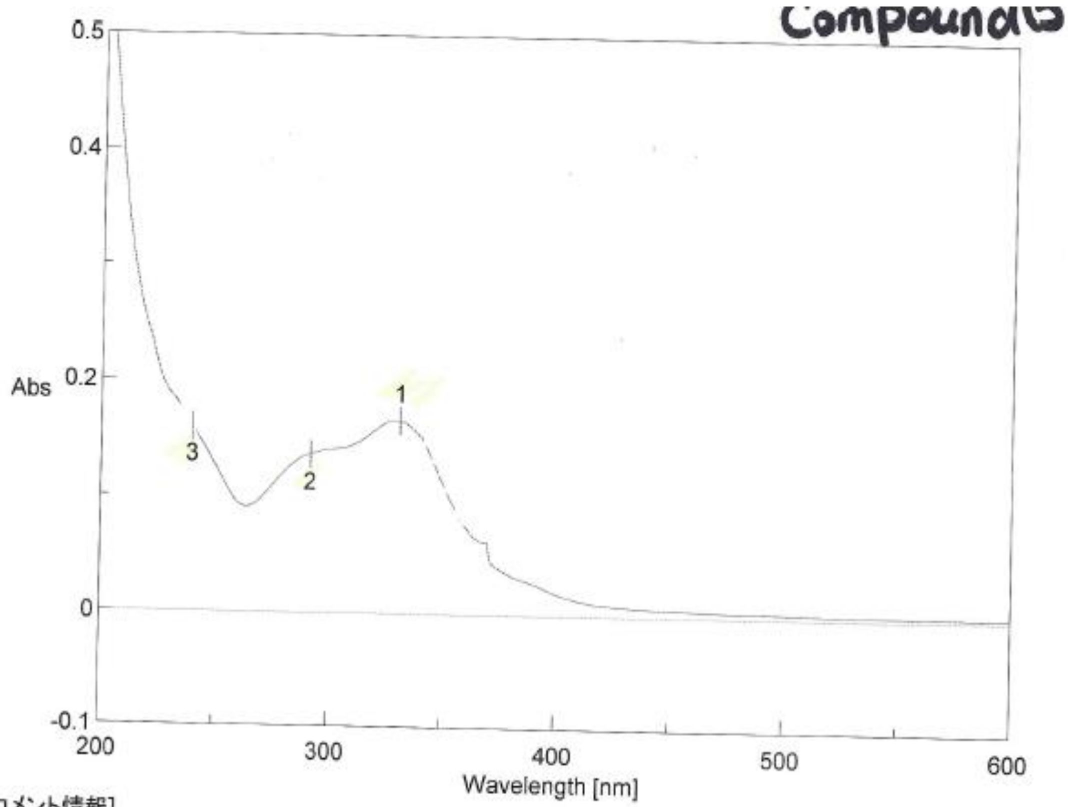

[コメント情報]

試料名	
コメント	
測定者	
所属	
会社	HU

[データ情報]

作成日時	2018/07/02 19:25
データタイプ	等間隔データ
横軸	Wavelength [nm]
縦軸	Abs
スタート	600 nm
エンド	200 nm
データ間隔	1 nm
データ数	401

[測定情報]

装置名	V-630
機種名	V-630
シリアル番号	A042161148
測定日時	2018/07/02 19:25
測光モード	Abs
測定範囲	600 - 200 nm
データ取込間隔	1 nm
UV/Vis バンド幅	1.5 nm
レスポンス	Fast
走査速度	400 nm/min
光源切換	340 nm
光源	D2/WI
フィルタ切換	ステップ
補正	ベースライン

[ピーク検出結果]

No.	位置	強度	No.	位置	強度
1	331	0.16674	2	292	0.136832
3	240	0.159461			
